# Protective Effects of *Pterostilbene* on Lipopolysaccharide-Induced Acute Lung Injury in Mice by Inhibiting NF-κB and Activating Nrf2/HO-1 Signaling Pathways

**DOI:** 10.3389/fphar.2020.591836

**Published:** 2021-01-29

**Authors:** Yong Zhang, Zhen Han, Aimin Jiang, Di Wu, Shuangqiu Li, Ziyi Liu, Zhengkai Wei, Zhengtao Yang, Changming Guo

**Affiliations:** ^1^ College of Veterinary Medicine, Jilin University, Changchun, China; ^2^ College of Life Sciences and Engineering, Foshan University, Foshan, China

**Keywords:** pterostilbene, lipopolysaccharide, acute lung injury, inflammatory response, oxidative stress

## Abstract

Pterostilbene (PTER) is a kind of stilbene compound with biological activity isolated from plants such as red sandalwood, blueberry and grape. It has anti-tumor, anti-bacterial, anti-oxidation and other pharmacological activities. However, the underlying mechanism of the protective effect of PTER on lipopolysaccharide (LPS)-induced acute lung injury (ALI) remained not clarified. In this study, LPS was used to establish a mouse model of ALI. Bronchoalveolar lavage fluid (BALF) was collected for inflammatory cells, and the wet-to-dry weight ratio of the lungs was measured. The activities of myeloperoxidase (MPO), antioxidant indexes such as superoxide dismutase (SOD), catalase (CAT), glutathione peroxidase (GSH-Px) and oxidation index such as malondialdehyde (MDA) in lung tissues of mice were measured by the corresponding kits. The levels of Cyclooxygenase-2 (COX-2), inducible nitric oxide synthase (iNOS), TNF-α, IL-6 and IL-1β in lung tissues of mice were detected by quantitative real-time polymerase chain reaction (qRT-PCR). The activities of Nrf2, HO-1, p-p65 and *p*-IκB were determined by western blotting. The results showed that the model of LPS-induced ALI was successfully replicated, and it was found that PTER could significantly improve the pathological degree of ALI such as sustained the integrity of the lung tissue structure, alleviated pulmonary interstitial edema and alveolar wall thickening, reduced infiltrated inflammatory cells. PTER could decrease the number of inflammatory cells and obviously inhibit the increase of W/D ratio caused by LPS. PTER could also significantly reduce LPS-induced MPO and MDA, and increase LPS-decreased SOD, CAT and GSH-Px in the lungs. In addition, it was also found that PTER has the ability to decrease LPS-induced production of COX-2, iNOS, TNF-α, IL-6 and IL-1β. The underlying mechanism involved in the protective effect of PTER on ALI were via activating Nrf2 and HO-1, and inhibiting the phosphorylation of p65 and IκB. These results suggested that PTER can protect LPS-induced ALI in mice by inhibiting inflammatory response and oxidative stress, which provided evidence that PTER may be a potential therapeutic candidate for LPS-induced ALI intervention.

## Introduction

Acute lung injury (ALI) is a respiratory disease characterized by a large number of inflammatory cell infiltration, damage to alveolar epithelial cells and capillary endothelial cells, destruction of alveolar structure, pulmonary interstitial edema, marked thickening of alveolar walls, and acute hypoxic respiratory insufficiency. ALI can be caused by many factors including pulmonary infection caused by viruses, bacteria, fungi, inhalation injury caused by high concentration of oxygen and other harmful gases, seawater and other liquids, lung and chest wall trauma, sepsis and so on (Confalonieri et al., 2017; Matthay et al., 2017). If the condition is aggravated, it can further develop into Acute respiratory distress syndrome (ARDS) (Ware and Matthay, 2000). Due to the complex pathogenesis of the disease, it has not yet been fully elucidated, and there is no effective prevention and treatment method. For human patients, respiratory support technology (mainly including: small tidal volume ventilation, positive end expiratory pressure, prone position ventilation, high frequency oscillatory ventilation, extracorporeal lung membrane technology and so on) is currently the most important treatment for ALI in clinical practice (Amato et al., 1998). Some drugs, such as glucocorticoids and ulinastatin, have therapeutic effects on ALI, but they are not widely recommended in clinic due to more adverse reactions (Liu et al., 2013). For livestock and poultry, ALI is mostly caused by dirty and humid environment, mildew, dirty air, overcrowding and poor ventilation in livestock houses. The morbidity and mortality of livestock and poultry remain high, causing significant economic losses to the livestock industry, and seriously hindering the development of the livestock industry.

Nuclear factor-κB (NF-κB), as a transcription factor in organism, plays an important role in physiological and pathological processes, such as regulating inflammatory response and apoptosis (Wei et al., 2015; Wang J. et al., 2019). For example, classical inflammatory cytokines, TNF-α, IL-6 and IL-1β are regulated by NF-κB. Cyclooxygenase-2 (COX-2) and inducible nitric oxide synthase (iNOS) interact with and interact with NF-κB. Activation of the NF-κB signaling pathway can induce the expression of COX-2 and iNOS. These induced gene products can further participate in inflammation and immune response, and play an important role in physiological and pathological conditions (Fan et al., 2018). Lots of experiments have confirmed that under the stimulation of inflammatory factors such as lipopolysaccharide (LPS), NF-κB can be transferred from cytoplasm to nucleus, from inactive state to active structure, thus initiating gene transcription of various inflammatory mediators and chemokines at the transcriptional level, thus intensifying the degree and duration of inflammation (Huang et al., 2016). Nrf2 is a key factor in oxidative stress. Its activation energy regulates a variety of downstream antioxidant enzymes (such as HO-1, SOD, CAT, and GSH-Px) to eliminate excessive free radicals in the body, thereby reducing the degree and duration of oxidative stress (Zhao et al., 2014). Therefore, ALI can be prevented or treated in both anti-oxidative stress and anti-inflammatory by activating Nrf2 and inhibiting NF-κB signaling pathway.

**FIGURE 1:**
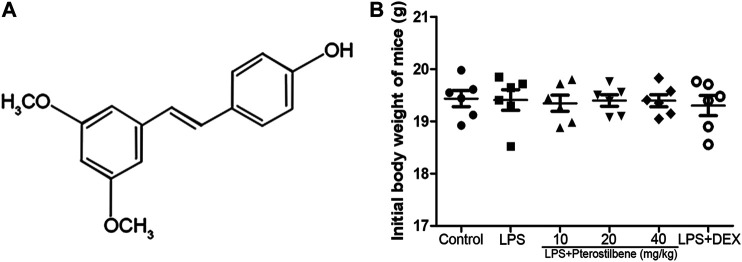
**(A)** Chemical structure of PTER. **(B)** Initial weight of mice.

Pure natural plant products have attracted wide attention for their remarkable efficacy and relatively low toxicity in the treatment of ALI. Therefore, the treatment of ALI with pure natural plant products is a valuable research direction and has great application prospects. *Pterostilbene* (3,5-Dimethoxy-4′-hydroxystilbene; Figure 1A) is a kind of stilbene compound extracted from small berries such as blueberries and grapes (Wang and Sang, 2018). It has been found that PTER has many biological effects such as anti-inflammatory (Paul et al., 2009) and anti-oxidative (Perecko et al., 2010) effects. Previous studies have shown that PTER and its compounds have protective effects against LPS-induced ALI (Park et al., 2018). However, the underlying mechanism involved in the protective effect of PTER on ALI are not completely clear. This study was designed to investigate the effects of PTER on LPS-induced ALI and to elucidate its molecular mechanisms.

## Materials and Methods

### Chemicals and Reagents

PTER (SP9570, purity ≥98%) was purchased from Beijing Solarbio Science and Technology Co., Ltd. (Beijing, China). LPS (L2880, purity >97%) was purchased from the Sigma Chemical Co. (St. Louis, MO, United States). MPO (A044-1–1), SOD (A001-3–2), CAT (A007-1–1), GSH-Px (A005-1–2) and MDA (A003-1–2) Assay Kit and were purchased from Nanjing Jiancheng Bioengineering Institute (Nanjing, China). TNF-α, IL-6, IL-1β, COX-2 and iNOS primers were purchased from Sangon Biotech Co., Ltd. (Shanghai, China). p-p65 rabbit polyclonal antibody (A13599) was acquired from Boster Biological Technology co.Itd. p65 rabbit polyclonal antibody (BS9879M), *p*-IκB rabbit polyclonal antibody (BS4105), IκB rabbit polyclonal antibody (BS3601), Nrf2 rabbit polyclonal antibody (BS1258) and HO-1 rabbit polyclonal antibody (BS6626) were acquired from Bioworld Technology, Inc (Minnesota, United States). Actin-β polyclonal antibody (YT0099) was acquired from ImmunoWay Biotechnology Company (Plano, TX, United States). All other chemicals were at the reagent level.

### Animals

Male BALB/c mice (4 weeks old; weight 18–20 g) were obtained from Liaoning Changsheng Biotechnology Co., Ltd (Liaoning, China). The initial weight of mice was shown in Figure 1B. Before the experiment, all mice were kept in a controlled environment with ambient temperature at 24 ± 1°C and humidity at 60 ± 5%, and a 12 h light/dark cycle was guaranteed. The mice were free to eat and drink, and adapted to the environment for one week. All the animal experiments were performed in accordance with the Animal Welfare and Research Ethics Committee at Jilin University (approval ID 20111106–2).

### Establishment of Acute Lung Injury in Mice

All the mice were randomly divided into six groups (*n* = 6 per group). The six groups are Control group, LPS-induced ALI model group, LPS + PTER (10, 20, and 40 mg/kg) groups and LPS + DEX group. All the mice were fasted for 8 h, but they could drink water freely. Before the establishment of the model of LPS-induced ALI, the LPS + PTER (10, 20, and 40 mg/kg) groups were given corresponding PTER concentrations, the mice of the LPS + DEX group were given DEX (5 mg/kg) and the Control group and the LPS-induce ALI model group were given equal volume of 0.9% NaCl solution. All the above administration methods were intraperitoneal injection (i.p.). 1 h later, all groups of mice were inhaled with a little of ether to make them slightly anesthetized. Except for the Control group, 10 μg LPS in 50 μl of 0.9% NaCl solution were injected into the nasal cavity of all groups of mice. And the mice of the Control group were given 50 μl of 0.9% NaCl solution in the same way. After treating the mice with LPS for 7 h, the mice were sacrificed by bloodletting and their lung tissues were collected.

### Cell Count and Protein Concentration of Bronchoalveolar Lavage Fluid in Mice

After the mice were sacrificed, the skin of the larynx was cut to expose the trachea, the catheter was inserted into the trachea from the mouth, and the trachea and the catheter were tied tightly with a surgical thread. Slowly rinsed with PBS solution three times, 500 μl each time, 1.5 ml in total, the washing fluid was BALF, and then the collected BALF was centrifuged at 4°C 3 000 rpm for 10 min, the supernatant was used to determine the protein concentration by BCA method, and the resulting precipitate was resuspended in PBS solution. The number of total cells, neutrophils and macrophages was measured with a blood cell analyzer instrument (Japan Photoelectric MEK-7222 K Whole Blood Cell Analyzer).

### Measurement of Wet-To-Dry Ratio of the Lungs

The whole lungs were removed, washed with PBS solution for three times, and then the water on the surface of the lungs was dried with absorbent paper and weighed immediately, that is, the wet weight. Then they were put into the incubator, baked at 80°C for 48 h, and then weighed again, that is, the dry weight, and then the ratio of wet weight to dry weight (W/D) was calculated.

### Histopathological Analysis

The right lungs were removed and placed in 10% formaldehyde solution and fixed for 24 h. Then, the lung tissues were treated with different concentrations of alcohol to dehydrate and wrapped in paraffin. Finally, the obtained lung tissue sections were stained with hematoxylin and eosin, and the histopathological changes of the lung tissues were observed with an optical microscope, and the images were collected.

### Superoxide Dismutase, Catalase, Glutathione Peroxidase, and Malondialdehyde Analysis

According to the manufacturer's protocols, the GSH-Px, CAT, SOD and MDA contents in lung tissues of mice were determined by GSH-Px, CAT, SOD and MDA assay kits.

### Quantitative Real-Time Polymerase Chain Reaction Analysis Cytokines in Lung Tissue

This experiment was designed to detect the expression of RNA of COX-2, iNOS, TNF-α, IL-6 and IL-1β in lung tissue as previous described (Wei et al., 2015). Total RNA was extracted from lung tissues of mice in each group by Trizol reagent. Then the extracted RNA was reverse transcribed into cDNA. qRT-PCR was carried out with a 7,500 real-time PCR system (Applied Biosystems, Carlsbad, CA). Primers were obtained from Sangon Biotech Co., Ltd. (Shanghai, China) and were listed in Table 1.

**TABLE 1:** Primers used in this study.

Primer name	Nucleotide sequence (5’-3’)
TNF-α forward	ACG​GGC​TTT​ACC​TCA​TCT​ACT​C
TNF-α reverse IL-6 forward IL-6 reverse	GCTCTTGATGGCAGACAGG AGTTGTGCAATGGCAATTCTGA CCC​CAG​CAT​CGA​AGG​TAG​A
IL-1β forward	AGG​TGG​TGT​CGG​TCA​TCG​T
IL-1β reverse COX-2 forwad COX-2 reverse iNOS forward iNOS reverse	GCTCTCTGTCCTGGAGTTTGC ATTCCAAACCAGCAGACTCATA CTTGAGTTTGAAGTGGTAACCG TGCCACGGACGAGACGGATAG CTCTTCAAGCACCTCCAGGAACG
GAPDH forward	TCA​ACG​GGA​AGC​TCA​CTG​G
GAPDH reverse	CCCCAGCATCGAAGGTAGA

### Western Blot Analysis

According to the manufacturer's protocols as previous described (Wei et al., 2018b; Liu et al., 2019; Wei et al., 2019), the total protein in the lung tissues were extracted with tissue total protein lysis buffer. The concentration of total protein was determined by BCA method. The obtained total protein samples were separated by SDS-polyacrylamide gel electrophoresis (SDS-PAGE) and transferred to polyvinylidene difluoride (PVDF) membranes. Then the PVDF membranes were put into TBST solution containing 5% skimmed milk powder and blocked for 3 h at room temperature. Then the PVDF membranes were incubated overnight with the corresponding antibody at 4°C. The PVDF membranes were washed with TBST solution and then incubated at room temperature for 2 h with appropriate horseradish peroxidase (HRP)-conjugated secondary antibodies. Then the PVDF membranes were washed with TBST solution. Finally, the PVDF membranes were treated with Immobilon Western Chemiluminescent HRP Substrate, the proteins were observed by enhanced chemiluminescence (ECL) under Western Blotting Detection System (Amersham Life Science, United Kingdom). We used ImageJ software to analyze strips.

### Statistical Analysis

The experimental data were analyzed by GraphPad Prism 5.0 software, and the results were expressed by mean ± SEM (Standard Error of Mean). In data analysis, one-way ANOVA was used to compare the data between groups, and LSD was used to make multiple comparisons. *p* < 0.05 or *p* < 0.01 showed significant or extremely significant statistical differences, respectively.

## Results

### Pterostilbene Reduced Lipopolysaccharide-Induced the Number of Inflammatory Cells and Protein Concentration in Bronchoalveolar Lavage Fluid

**FIGURE 2:**
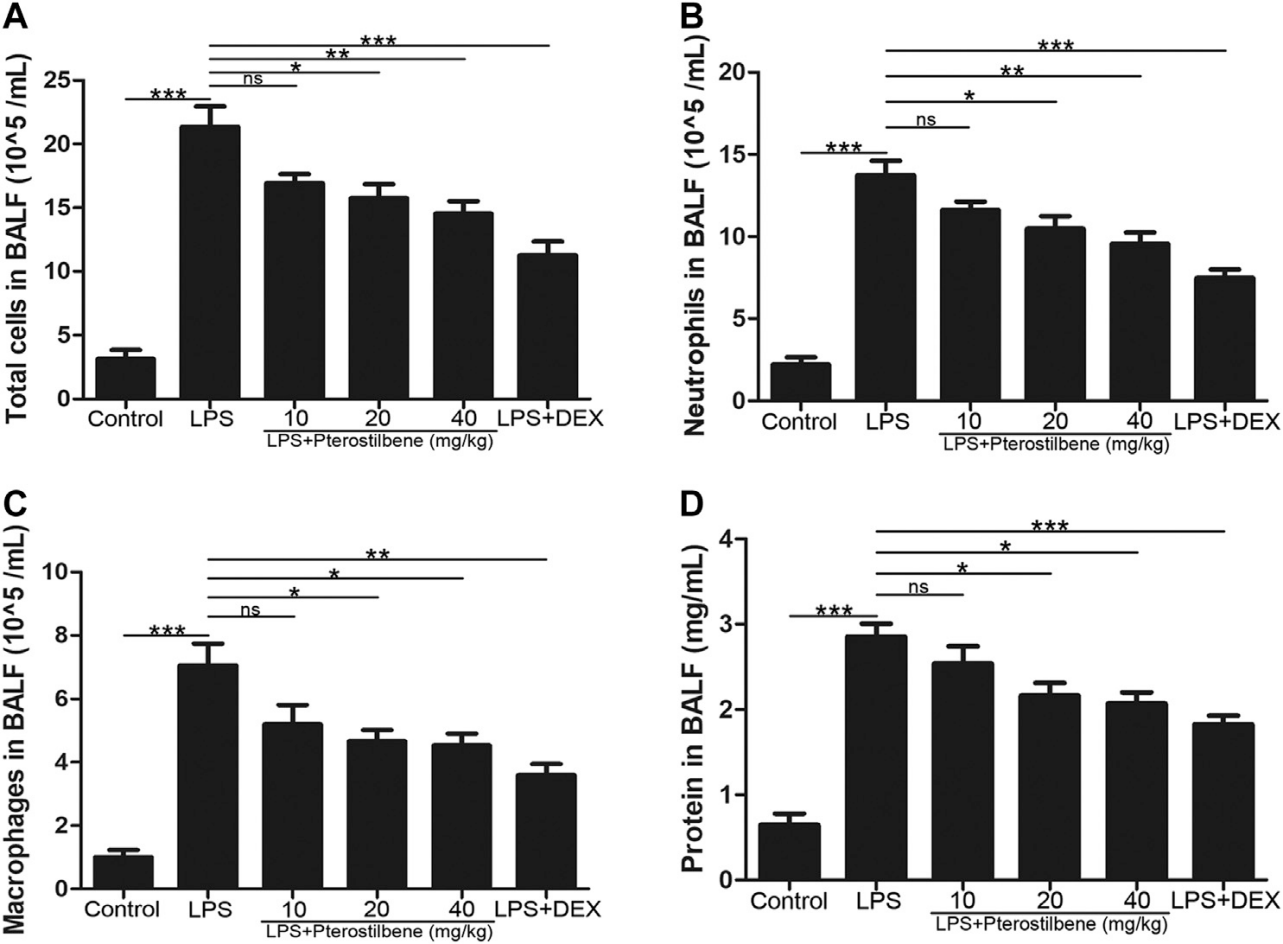
PTER reduced LPS-induced the number of inflammatory cells and protein concentration in BALF. **(A)** Total cells in BALF. **(B)** Neutrophils in BALF. **(C)** Macrophages in BALF. **(D)** Protein concentration in BALF. Values are presented as mean ± SEM (*n* = 6). *p* values of <0.05 were considered significant (*p < 0.05; **p < 0.01; ***p < 0.001; “ns” means not significant).

In order to confirm the effect of PTER on inflammatory cells in BALF with LPS-induced ALI, BALF was collected to count the number of total cells, neutrophils and macrophages. Compared with control group, the number of total cells (Figure 2A), neutrophils (Figure 2B), and macrophages (Figure 2C) in BALF in the LPS-induced model group increased significantly, while PTER pretreatment reduced the infiltration of these inflammatory cells into the lung tissue. Moreover, compared with control group, the protein concentration in BALF in the LPS-induced model group increased significantly, while PTER pretreatment reduced the protein concentration in BALF (Figure 2D). These results suggested that PTER could inhibit the migration of inflammatory cells to lung tissue, thereby reducing LPS-induced inflammatory responses of in ALI.

### Pterostilbene Reduced Lipopolysaccharide–Increased W/D Ratio of the Lungs

**FIGURE 3:**
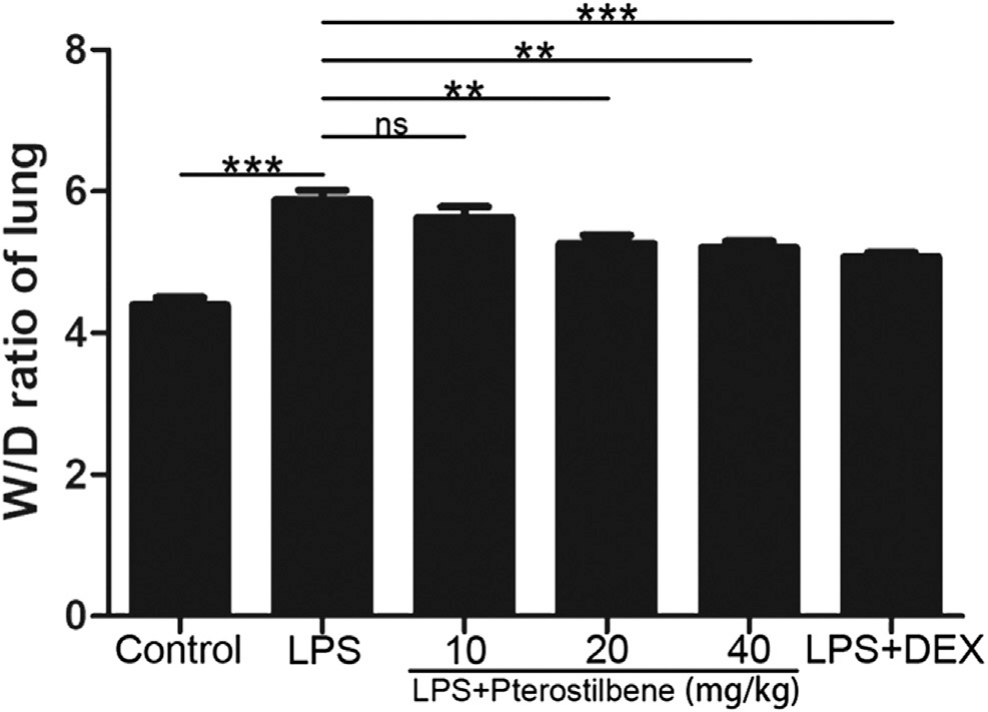
PTER reduced LPS‐increased W/D ratio of the lungs. Values are presented as mean ± SEM (*n* = 6). *p* values of <0.05 were considered significant (**p < 0.01; ***p < 0.001; “ns” means not significant).

The W/D ratio of the lungs can reflect the degree of edema in LPS-induced ALI model in mice. As shown in Figure 3, compared with control group, the W/D ratio increased significantly after LPS stimulation. After DEX treatment, the increase in W/D ratio caused by LPS stimulation was significantly inhibited. When the PTER concentration was 10 mg/kg, it had no obvious inhibitory effect on the increase of W/D value caused by LPS, and when the concentration of PTER increased to 20 and 40 mg/kg, it could obviously inhibit the increase of W/D ratio caused by LPS. The result showed that PTER could significantly reduce the degree of the lungs edema in LPS-induced ALI model in mice.

### Pterostilbene Attenuated Lipopolysaccharide-Induced Histopathological Changes in the Lungs

**FIGURE 4:**
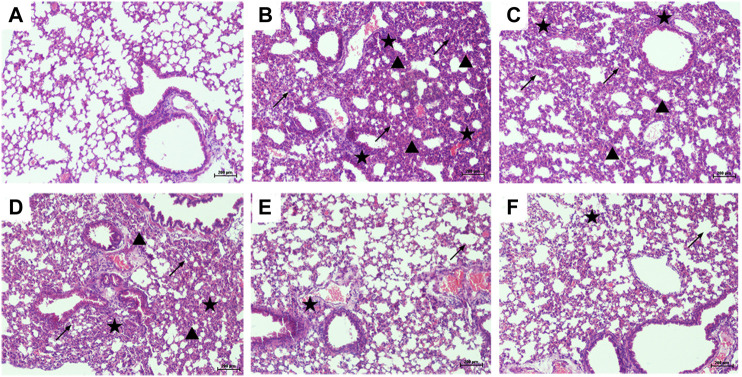
PTER attenuated LPS-induced histopathological changes in the lungs (100×). Mice treated with **(A)** 0.9% NaCl solution **(B)** LPS + 0.9% NaCl solution **(C)** LPS + PTER 10 mg/kg **(D)** LPS + PTER 20 mg/kg **(E)** LPS + PTER 40 mg/kg **(F)** LPS + DEX 5 mg/kg. The stars show a large number of inflammatory cells infiltrating, the triangles show thickening of alveolar septum, and the arrows show destruction of alveolar structure.

The lung tissues of mice were stained with H&E to observe the pathological changes, and the protective effect of PTER on LPS-induced ALI was revealed intuitively. Compared with control group (Figure 4A), the lung tissue of mice in LPS-induced ALI model group (Figure 4B) showed obvious and unique pathological changes, such as smaller alveolar cavity, alveolar structure destruction, thicker alveolar septum, congestion and edema of alveolar wall, and a large number of inflammatory cell infiltration. Compared with the LPS-induced ALI model group, the lung tissue structure of mice in LPS + PTER (10, 20, and 40 mg/kg) group (Figures 4C–E) and LPS + DEX group (Figure 4F) tended to be normal, pulmonary interstitial edema and alveolar wall thickening were alleviated, inflammation was reduced, and inflammatory cells were decreased. These results suggest that PTER has a protective effect on LPS-induced ALI in a dose-dependent manner.

### Pterostilbene Reduced Lipopolysaccharide-Induced Myeloperoxidase in the Lungs

**FIGURE 5:**
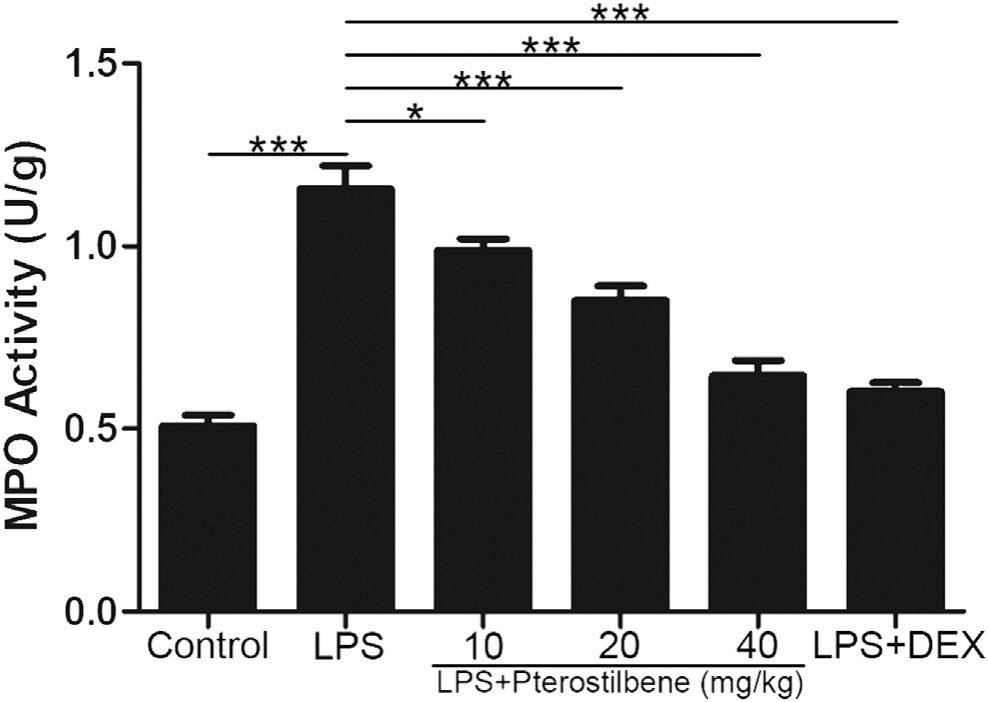
PTER reduced LPS-induced MPO in the lungs. Values are presented as mean ± SEM (*n* = 6). *p* values of <0.05 were considered significant (*p < 0.05; ***p < 0.001).

MPO activity is an effective indicator of neutrophil influx into lung tissue. As shown in Figure 5, LPS significantly increased MPO activity in the lungs of mice. Compared with LPS model group, PTER (10, 20, and 40 mg/kg) and DEX decreased MPO activity. These data suggested that PTER can reduce the activity of MPO in the lungs of mice in a dose-dependent manner.

### Pterostilbene Increased Superoxide Dismutase, Catalase, Glutathione Peroxidase, and Induced Malondialdehyde in the Lungs

**FIGURE 6:**
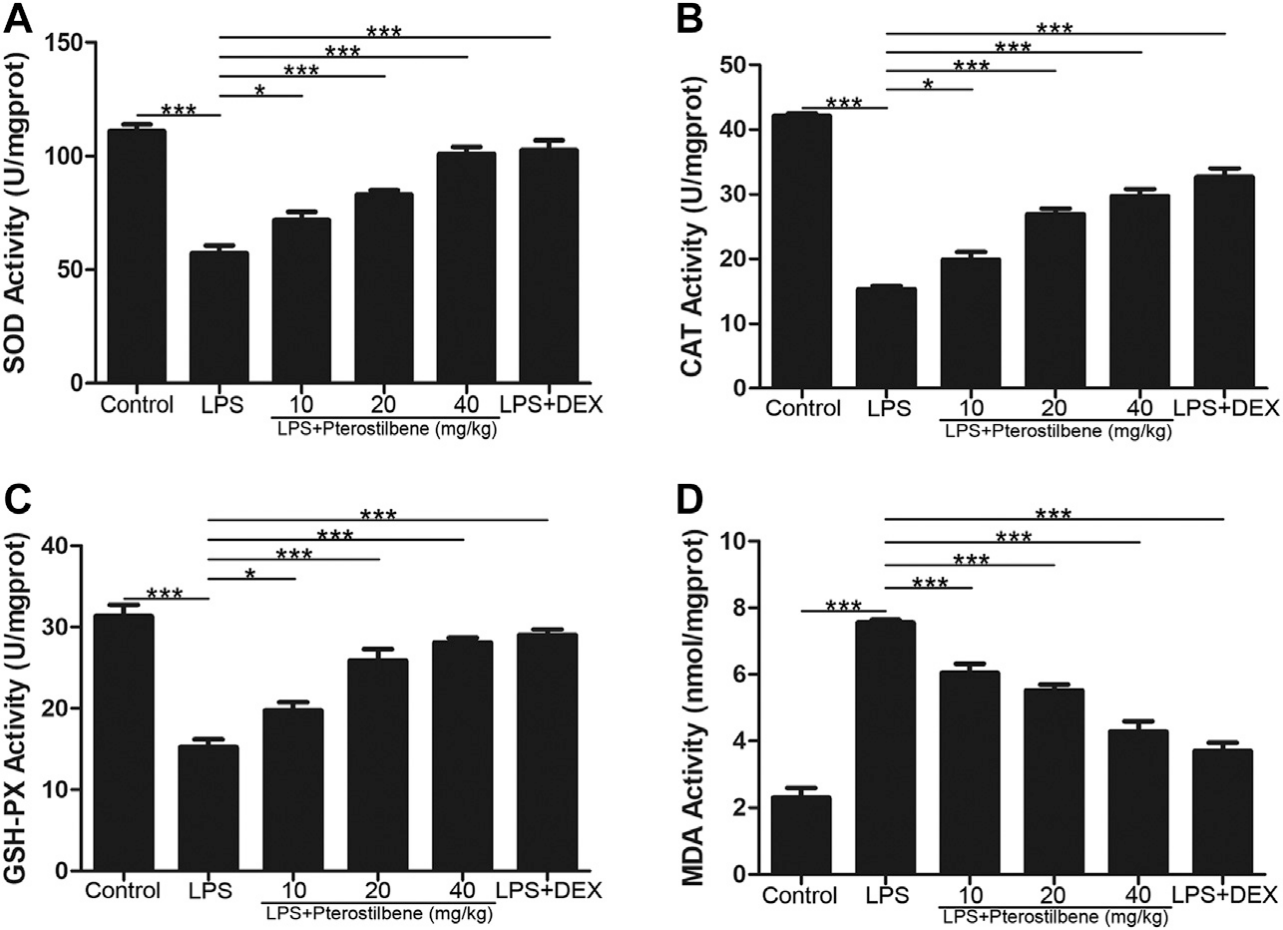
PTER increased SOD, CAT, and GSH-Px and reduced MDA in the lungs. **(A)** SOD activity of lung tissues in mice. **(B)** CAT activity of lung tissues in mice. **(C)** GSH-Px activity of lung tissues in mice **(D)** MDA activity of lung tissues in mice. Values are presented as mean ± SEM (*n* = 6). *p* values of <0.05 were considered significant (*p < 0.05; ***p < 0.001).

MDA is an important indicator of oxidative stress, and SOD, CAT, and GSH-Px are important indicators of antioxidant stress. Through the determination of these indicators, it was verified that PTER can protect LPS-induced ALI in mice through anti-oxidative stress. The results showed that MDA increased significantly in mice exposed to LPS. MDA levels in LPS + PTER (10, 20, and 40 mg/kg) group and LPS + DEX group were significantly lower than those in LPS-induced ALI model groups (Figure 6D). In addition, LPS stimulation reduced the activities of SOD, CAT and GSH-Px. Treatment with PTER (10, 20 and 40 mg/kg) and DEX significantly increased SOD, CAT and GSH-Px levels in lung tissue of mice (Figures 6A–C). The results showed that PTER could protect mice from LPS-induced ALI by reducing MDA, increasing SOD, CAT and GSH-Px in a dose-dependent manner.

### Pterostilbene Reduced Lipopolysaccharide-Induced COX-2, iNOS, TNF-α, IL-6 and IL-1β in the Lungs

Inflammatory cytokines and oxidative stress are involved in the initiation and expansion of inflammation and persist in LPS-induced ALI. We measured the levels of cytokines in lung tissue of mice by qRT-PCR.

**FIGURE 7:**
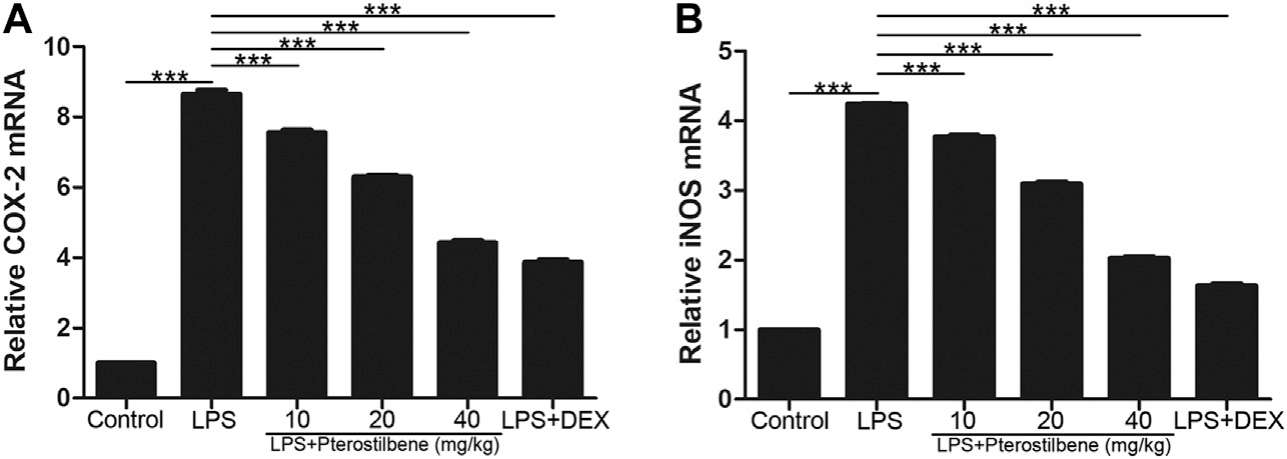
PTER reduced LPS-induced COX-2 and iNOS in the lungs. The relative **(A)** COX-2 and **(B)** iNOS expressions of lung tissues in mice were evaluated by qRT-PCR. Values are presented as mean ± SEM (*n* = 6). *p* values of <0.05 were considered significant (***p < 0.001).

In terms of oxidative stress, the result showed that compared with control group, LPS significantly increased the production of two important pro-inflammatory enzymes COX-2 and iNOS in lung tissue. In addition, PTER significantly inhibited COX-2 and iNOS induced by LPS (Figures 7A, B), which confirmed its good anti- oxidative stress activity *in vivo*. The results showed that PTER could protect mice from LPS-induced ALI by inhibiting the release of COX-2 and iNOS in a dose-dependent manner.

**FIGURE 8:**
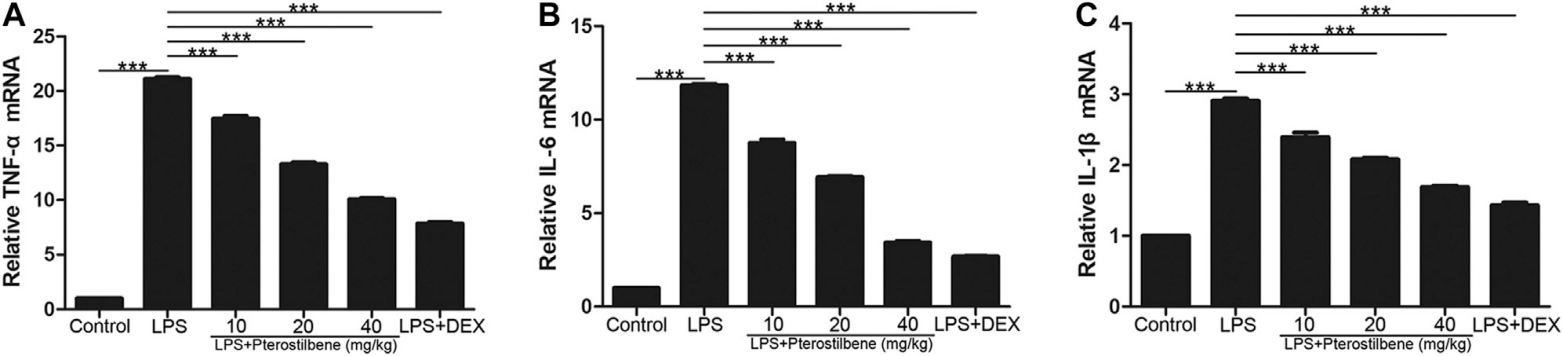
PTER reduced LPS-induced TNF-α, IL-6 and IL-1β in the lungs. The relative **(A)** TNF-α **(B)** IL-6 and **(C)** IL-1β expressions of lung tissues in mice were evaluated by qRT-PCR. Values are presented as mean ± SEM (*n* = 6). *p* values of <0.05 were considered significant (***p < 0.001).

In terms of inflammation, the result showed that compared with control group, LPS significantly increased TNF-α, IL-6 and IL-1β in lung tissue. On the other hand, PTER significantly reduced the secretion of cytokines TNF-α, IL-6 and IL-1β induced by LPS (Figures 8A–C), which confirmed its good anti-inflammatory activity *in vivo*. The results showed that PTER could protect mice from LPS-induced ALI by inhibiting the release of TNF-α, IL-6 and IL-1β in a dose-dependent manner.

### Pterostilbene Inhibited the Protein Expressions of NF-κB Signaling Pathway in the Lungs

**FIGURE 9:**
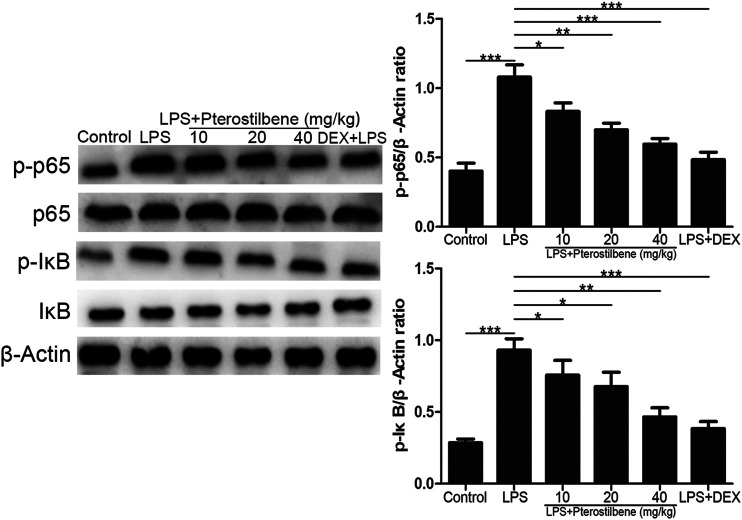
PTER inhibited the protein expressions of NF-κB signaling pathway in the lungs. Values are presented as mean ± SEM (*n* = 6). *p* values of <0.05 were considered significant (*p < 0.05; **p < 0.01; ***p < 0.001).

NF-κB signaling pathway has long been considered as a typical inflammatory signaling pathway. As shown in Figure 9, compared with control group, LPS-induced ALI model group significantly promoted the expression of p-p65 and *p*-IκB. Compared with LPS-induced ALI model group, LPS + PTER (10, 20, and 40 mg/kg) group and LPS + DEX group significantly inhibited the expression of p-p65 and *p*-IκB, and the degree of inhibition increased with the increase of PTER concentration in a dose-dependent manner. The results showed that the protective effect of PTER on LPS-induced ALI in mice was related to inhibition of p-p65 and *p*-IκB expression.

### Pterostilbene Activated the Protein Expressions of Nrf2/HO-1 Signaling Pathway in the Lungs

**FIGURE 10:**
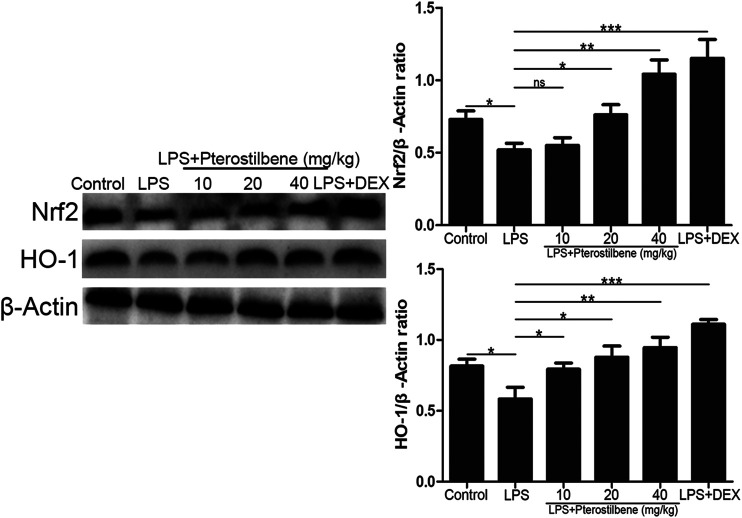
PTER activated the protein expressions of Nrf2/HO-1 signaling pathway in the lungs. Values are presented as mean ± SEM (*n* = 6). *p* values of <0.05 were considered significant (*p < 0.05; **p < 0.01; ***p < 0.001; “ns” means not significant).

Nrf2 is an activator of the antioxidant response element ARE and is considered to be the core transcription factor regulating the antioxidant stress response. As shown in Figure 10, compared with control group, the expression of Nrf2 and HO-1 in lung tissue of LPS-induced ALI model group was significantly lower. Compared with LPS-induced ALI model group, the expression of Nrf2 and HO-1 in lung tissue of LPS + PTER (10, 20, and 40 mg/kg) group and LPS + DEX group increased significantly in a dose-dependent manner with the increase of PTER concentration. The results showed that the protective effect of PTER on LPS-induced ALI in mice was related to up-regulation of Nrf2 and HO-1 expression.

## Discussion

ALI is a serious respiratory disease in animal feeding process, which seriously threatens the life safety of sick livestock and poultry. LPS is a component of the cell wall of Gram-negative bacteria and it is also an important stimulator triggering inflammation in the body (Bhattacharyya et al., 2004). Intranasal instillation of LPS is a classical and widely used method to construct ALI model (Hsu et al., 2015; Huang et al., 2015). This method can better simulate the pathological process of ALI, control the degree of ALI, only cause local severe injury, and will not cause systemic inflammatory response and organ failure (Santos et al., 2018). Recently, a study has confirmed that PTER can protect female mice in this ALI model (Yang et al., 2020). However, the protective effect of PTER in male mice has not been fully investigated. Therefore, adult male mice are used in this study to establish ALI model and evaluate the protective effect of *pterostilbene* in male mice.

Dexamethasone (DEX) is a synthetic glucocorticoid that has been widely used to reduce various inflammatory reactions and has significant effects. However, under normal circumstances, the hormone content in the body is extremely small, which can regulate metabolism, growth and development and other physiological activities. Once the body ingests hormones for a long time, it will inevitably have some toxic and side effects on the body. For example, long-term use of DEX can easily lead to osteoporosis, abnormal mental symptoms, disorder of material metabolism and water and salt metabolism of the body. After DEX is stopped, it will also produce such adverse symptoms as anorexia, vomiting, fatigue, muscle and joint pain. PTER is derived from small berry plants and belongs to natural structural compounds. It has anti-inflammatory and anti-oxidant effects, and has the advantages of small toxic and side effects, low drug resistance, and not easy to remain. Previous experiments had proved that PTER had protective effect on ALI (Park et al., 2018), but the underlying mechanism is still unclear. The purpose of this study is to reveal the specific protective mechanism of PTER on ALI. At the same time, this experiment also set up LPS + DEX group, compared the protective effect of PTER with that of DEX, in order to evaluate the protective effect of PTER on LPS-induced ALI. With the development of new drugs, it is essential to appropriately transfer the dosage of the drugs from one animal species to another. In this study, we explored the protective mechanism of PTER on LPS-induced ALI in mice at these concentrations (10, 20, and 40 mg/kg). According to the concentrations of PTER in mice, we can calculate the effective concentrations of PTER in poultry, livestock and other economic animals, even in human body through the drug dose conversion formula between different animals, which can provide reference and basis for the use concentrations of PTER in veterinary and human clinical treatment of ALI, which has certain reference value and significance. For humans, the conversion formula is as follows: Human Equivalent Dose (HED) = Animal dose (mg/kg) × [Animal *Km*/Human *Km*]. *Km* factor = Animal weight/Animal body surface area. *Km* Mouse *Km* = 3, Human *Km* = 37 (Reagan-Shaw et al., 2008). In this study, the most effective concentration in mice is 40 mg/kg, so it can be calculated that HED = 40 mg/kg × 3/37 = 3.24 mg/kg. The formula is also applicable to the conversion of drug dosage between different animals.

In the process of drug research and development, its drug metabolism in the body is very important. Through the study of its metabolism, many pharmacokinetic data can be understood and obtained to prepare for large-scale clinical research. The chemical structure of *pterostilbene* contains two benzene rings and one conjugated double bond, which has good UV absorption. UV absorption detector is generally used for *in vitro* analysis, and MS method with higher sensitivity is selected for *in vivo* analysis. Shao et al. (2010) gave *pterostilbene* solution (200 mg/kg-1, DMSO) to mice by gavage. Urine was collected 24 h later. The metabolites of *pterostilbene* in mouse urine were studied by LC/APCI-MS/MS method. It has been found that nine new metabolites of *pterostilbene* have been identified by MSn which are sulfated or glucuronized metabolites, such as *pterostilbene* glucuronide, *pterostilbene* sulfate, mono-demethylated *pterostilbene* glucuronide, mono-demethylated *pterostilbene* sulfate, mono-hydroxylated *pterostilbene*, mono-hydroxylated *pterostilbene* glucuronide, mono-hydroxylated *pterostilbene* sulfate, and mono-hydroxylated *pterostilbene* glucuronide sulfate. Lin et al. (2009) conducted a pharmacokinetic study on intravenous and oral *pterostilbene* in SD rats and found that the half-life and clearance rate of *pterostilbene* intravenously were (96.6 ± 23.7) min and (37.0 ± 2.5) min. The bioavailability of the drug is (72.5 ± 4.7) %, which may be due to the first pass effect reducing the blood content of *pterostilbene*. Remsberg et al. (2008) analyzed the pharmacokinetics of *pterostilbene* in rats. After fasting for 12 h, the rats were injected intravenously with *pterostilbene* at 20 mg/kg. Regular blood samples are collected through intubation. *Pterostilbene* was quickly removed from the serum (t 1/2 = 1.73 h). Within 30 min, the concentration of *pterostilbene* dropped from about 100 μg/ml to about 2 μg/ml. Glucuronate appeared in the earliest sample, that is, 1 min, about 6 μg/ml. Glucuronic acid increases slightly at about 1–2 h, indicating enterohepatic circulation. The methods they used to determine the concentration of *pterostilbene* and its metabolites were high performance liquid chromatography tandem mass spectrometry (LC-MS/MS). But the conditions of our laboratory could not finish the experiments of levels of *pterostilbene* and its metabolites in plasma/serum/tissue (lungs), we only discussed it here.

LPS-induced ALI can cause pulmonary edema, the volume and weight of lung tissue increase, which is one of the main characteristics of acute lung injury (Zhang et al., 2009). The W/D value of lung tissue was calculated to objectively evaluate the degree of pulmonary edema. The higher the W/D value, the more serious the pulmonary edema. In addition, in LPS. In addition, in LPS induced ALI, a large number of neutrophils to the lung tissue and participate in the inflammatory reaction. By counting the total cells, neutrophils and macrophages in the BALF, it was more fully confirmed that PTER reduced the number of neutrophils in the inflammatory site, which indicated that PTER could inhibit the migration of neutrophils to lung tissue, thereby reducing the inflammatory injury of lung tissue.

ROS plays a dual role in the biological activities of organisms (Wei et al., 2018a; Wei et al., 2018c; Wang C. et al., 2019; Yan et al., 2020). In the low physiological state, it can be used as a signaling molecule to participate in processes such as cell division, apoptosis and immune response (Han et al., 2019). The removal of excess ROS by related enzymes maintains this relatively stable state. Under pathological conditions, ROS clearance is inhibited and causes accumulation in cells. Hydroxyl radicals can react with purine, pyrimidine and deoxyribose skeletons in DNA molecules, destroying DNA structure, and oxidative damage to unsaturated fatty acids Oxide MDA. ROS also oxidizes cysteine and methionine residues in protein structures, resulting in the formation of reversible disulfide bonds between protein sulfhydryl groups and GSH, affecting the binding of GSH to electrophiles. An oxidative stress reaction occurred. ROS in the body are mainly cleared by antioxidant enzymes, including SOD, CAT and GSH-Px. After being reduced to H_2_O_2_ by SOD, it continues to generate H_2_O under the action of CAT and GSH-Px, and is excreted from the body, thereby eliminating ROS and protecting the body from oxidative attack. SOD is the only enzyme in the body that can remove O_2_^−^. It plays an important role in cell protection. The level of oxidative stress can be reflected by these indicators: MPO is a marker of neutrophil aggregation, which itself and a large number of oxidants derived from MPO can cause tissue damage (Xie et al., 2018). MDA is a product of lipid peroxidation, which is often used to reflect the level of oxidative stress (Jiang et al., 2016). SOD, CAT and GSH-Px are three important antioxidants in the body, which will be consumed heavily under oxidative stress (Manca et al., 1991; Annapurna et al., 2013). Therefore, we measured the above indicators in the experiment. The results showed that PTER had good antioxidant activity in LPS-induced ALI model: PTER significantly inhibited the production of MPO and MDA in lung tissue of LPS-induced mice, and significantly increased the contents of SOD, CAT, and GSH-Px.

In response to oxidative stress damage, the body has developed a complex oxidative stress response system. One of the defense mechanisms is the antioxidant response element ARE, which is a *cis*-enhancing element in the upstream of phase II detoxification enzymes and a variety of antioxidant protein/enzyme genes. Recent studies have found that nuclear transcription-related factor Nrf2 is an activator of ARE, and is considered to be the core transcription factor regulating antioxidant stress response (Zhang et al., 2017). It is a receptor of exogenous toxic substances and oxidative stress. It is closely related to the occurrence and development of inflammation, respiratory diseases, malignant tumors, precancerous lesions and cardiovascular diseases (Kansanen et al., 2013; Milani et al., 2013). Normally, Nrf2 binds to Keap1, a specific receptor in the cytoplasm, in the form of heterodimer, which is activated under oxidative stress, decouples with Keap1, translocates into the nucleus, binds to ARE, and then activates the transcription of ARE-mediated target genes and promotes the release of SOD, CAT, and GSH-Px to increase cell resistance to oxidative stress (Kitaoka et al., 2013; Gan and Johnson, 2014). Many Nrf2-related target genes such as HO-1 are expressed in the lungs. HO-1, also known as heat shock protein 32, is an endogenous antioxidant enzyme that attracts much attention. Studies have shown that HO-1 and related products of heme metabolism can play an antioxidant stress role. Targeted activation of HO-1 can prevent the occurrence and development of ALI (Park et al., 2018). In this study, the expression of Nrf2 and HO-1 in lung tissue of mice 7 h after ALI model was established, and the role and signaling pathways of oxidative stress in the pathogenesis of ALI were analyzed. The results showed that the expression of Nrf2 and HO-1 in lung tissue of the LPS-induced model group was lower than that of the Control group. It is suggested that oxidative stress may reduce the expression of antioxidant protein HO-1 by interfering with Nrf2-ARE signaling pathway, thus reducing the resistance of lung cells to oxidative stress and leading to ALI.

Nuclear factor NF-κB signaling pathway has long been considered as a typical inflammatory signaling pathway. Because it regulates gene expression of inflammatory factors, chemokines and adhesion molecules, it is often used as a key target of anti-inflammatory drug intervention in experiments (Shen et al., 2009). NF-κB is widely distributed in organisms. It can regulate the growth, differentiation, inflammation and apoptosis of all cells. Numerous experiments have proved that the NF-κB signaling pathway can indeed regulate the production of pro-inflammatory cytokines and induce leukocyte aggregation (Li et al., 2018). Through feedback mechanism, it can continuously activate leukocytes to induce systemic inflammatory response, and directly affect the intensity and duration of inflammatory response. It has also been shown that the apoptotic function of NF-κB can prevent the spread of inflammation by promoting leukocyte apoptosis, maintaining the survival of epithelial cells and the integrity of mucosal barrier (Andonegui et al., 2002). Future research needs to assess the different roles of NF-κB and their respective cellular signaling pathways in different disease states, and to intervene effectively with this goal, which may open up new ideas for the treatment of inflammatory diseases. Inflammation is usually considered as the main adaptive response. In classical inflammatory signaling pathways, NF-κB can be stimulated by extracellular pathogenic factors, such as endotoxin (LPS), oxygen free radicals, radiation and other stimulating factors (Yang et al., 2012), which can induce the activation of NF-κB signaling pathway, change the conformation of NF-κB in resting state, dissociate from complex state, transfer from cytoplasm to nucleus, and correspond to it in nucleus. The specific binding of κB loci on the gene regulates the expression of genes related to inflammatory mediators (Perkins, 2007). After the activation of the NF-κB signaling pathway, it can enhance the transcription of TNF-α, IL-6 and IL-1β genes. With the increase in the production and release of TNF-α, IL-6 and IL-1β, the NF-κB signaling pathway is activated again, resulting in further amplification of the initial inflammatory signal, exacerbating body damage and microcirculation disorders, ALI can aggravate the conversion to ARDS. COX-2 and iNOS are two important signaling protein molecules in the NF-κB signaling pathway. Under physiological conditions, the activity of iNOS and COX-2 in most tissues is almost not expressed; both can be rapidly induced and expressed under pathological conditions. The promoter sequence of COX-2 contains a specific binding sequence of NF-κB, which can promote the transcription of COX-2 gene after binding to NF-κB. iNOS and COX-2 coordinate with each other, directly damage the DNA and protein of cells, and play an important role in activating NF-κB. At present, many *in vivo* and *in vitro* experiments have shown that PTER can play a powerful anti-inflammatory role by inhibiting the NF-κB signaling pathway, which can mediate the regulation of a variety of inflammatory mediators, including COX-2, iNOS, TNF-α, IL-6 and IL-1β and so on (Cichocki et al., 2008; Zhang and Zhang, 2016; Yao et al., 2018). It was found that p-p65 and *p*-IκB proteins in lung tissue of ALI mice were significantly higher than those in blank group. Inhibiting the activity of NF-κB signaling pathway by PTER could significantly reduce the degree of inflammatory reaction in lung tissue, indicating that controlling the NF-κB signaling pathway is an important link in preventing ALI and preventing disease progression. Therefore, inhibiting the NF-κB signaling pathway is a feasible way to prevent ALI.

In conclusion, our study has shown that PTER can significantly protect LPS-induced ALI in mice, significantly inhibit LPS-caused histopathological changes, the number of inflammatory cells in BALF, and the increase of W/D ratio. PTER can improve the antioxidant capacity of lung tissue and reduce the inflammatory cytokines induced by LPS, and play a protective in LPS-induced ALI through regulating Nrf2/HO-1 and NF-κB signaling pathways. All these results indicate that PTER may be a drug to prevent ALI and has broad prospects.

## Data Availability Statement

The original contributions presented in the study are included in the article/Supplementary Material, further inquiries can be directed to the corresponding authors.

## Ethics Statement

The animal study was reviewed and approved by Animal Welfare and Research Ethics Committee at Jilin University.

## Author Contributions

YZ, ZH, AJ, DW, SL, and ZL assisted in carrying out the experiment. YZ wrote the manuscript. ZW, ZY, and CG helped with the design of experimental ideas and the revision of manuscripts.

## Funding

This work was funded by the National Key Research and Development Program (2016YFD0501203) and the National Natural Science Foundation of China (No. 31772721).

## Conflict of Interest

The authors declare that the research was conducted in the absence of any commercial or financial relationships that could be construed as a potential conflict of interest.
