# Supplementary figures and images for: Protective Effects of Pterostilbene on Lipopolysaccharide-Induced Acute Lung Injury in Mice by Inhibiting NF-κB and Activating Nrf2/HO-1 Signaling Pathways
Source: Front Pharmacol. 2021 Jan 29;11:591836. doi: 10.3389/fphar.2020.591836 (PMC7901969; doi:10.3389/fphar.2020.591836)

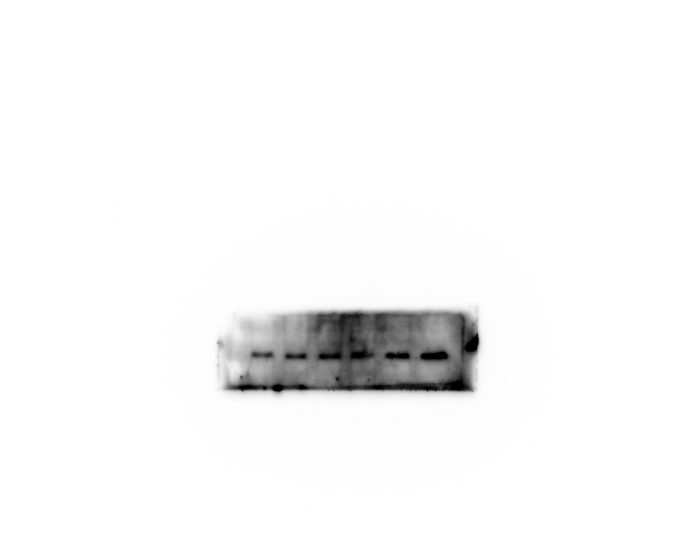

Supplement: Supplementary file 1 [file datasheet1.zip › The raw data of Western Blotting analysis/HO-1/HO-1(1).tif]

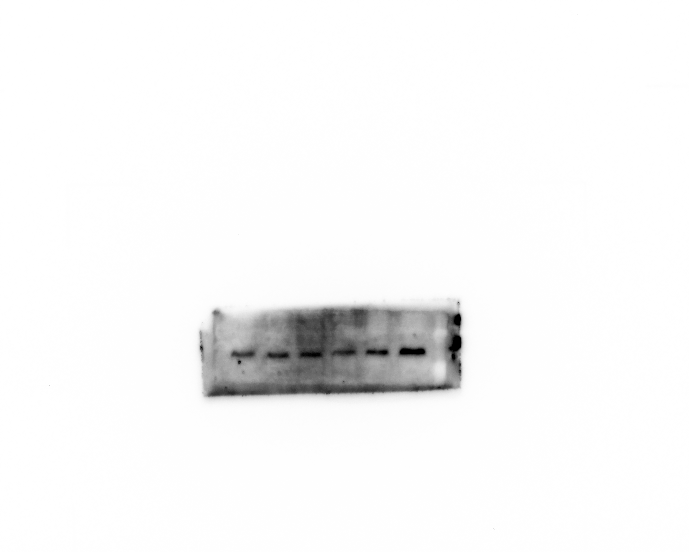

Supplement: Supplementary file 1 [file datasheet1.zip › The raw data of Western Blotting analysis/HO-1/HO-1(2).tif]

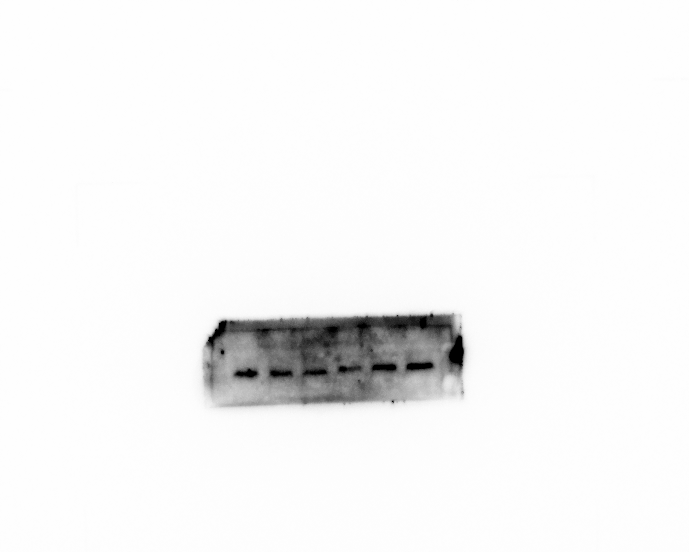

Supplement: Supplementary file 1 [file datasheet1.zip › The raw data of Western Blotting analysis/HO-1/HO-1(3).tif]

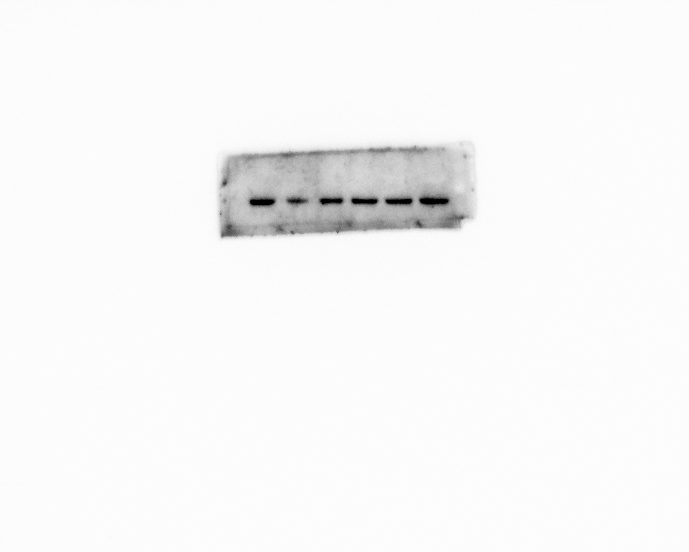

Supplement: Supplementary file 1 [file datasheet1.zip › The raw data of Western Blotting analysis/HO-1/HO-1(4).tif]

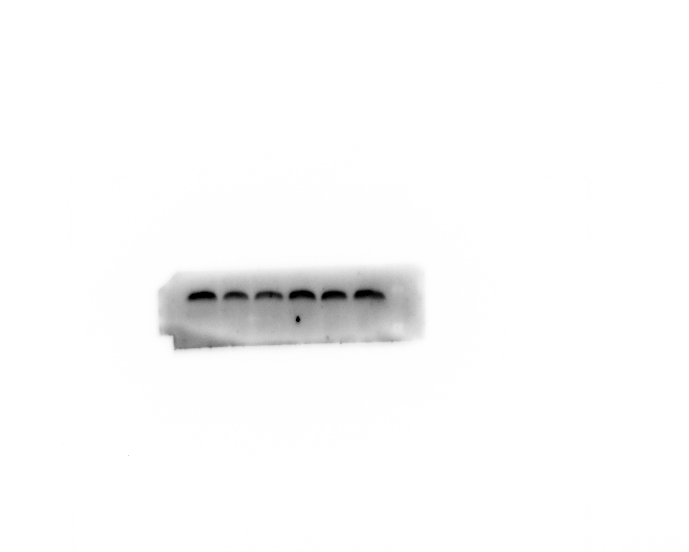

Supplement: Supplementary file 1 [file datasheet1.zip › The raw data of Western Blotting analysis/HO-1/HO-1(5).tif]

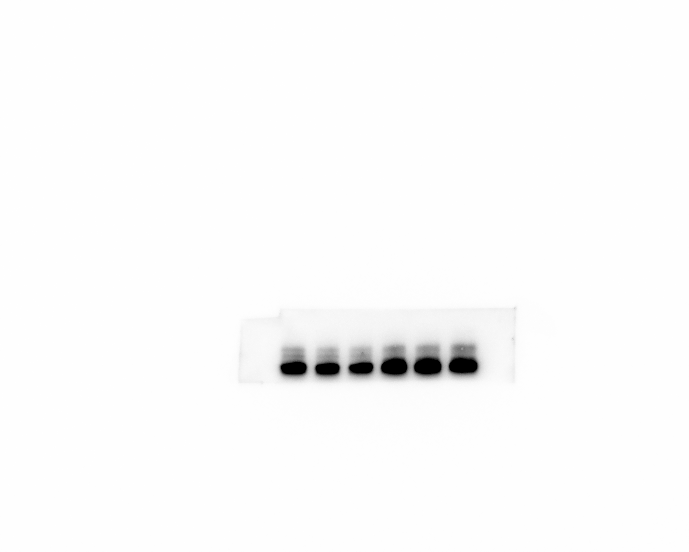

Supplement: Supplementary file 1 [file datasheet1.zip › The raw data of Western Blotting analysis/HO-1/HO-1(6).tif]

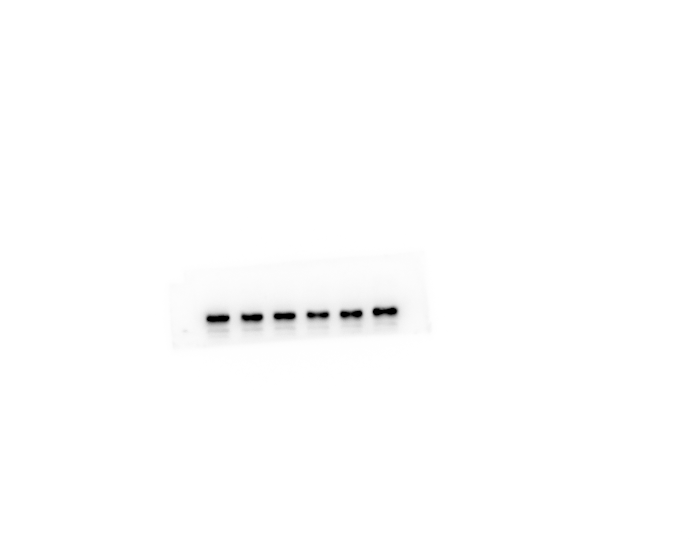

Supplement: Supplementary file 1 [file datasheet1.zip › The raw data of Western Blotting analysis/IκB/IκB-(1).tif]

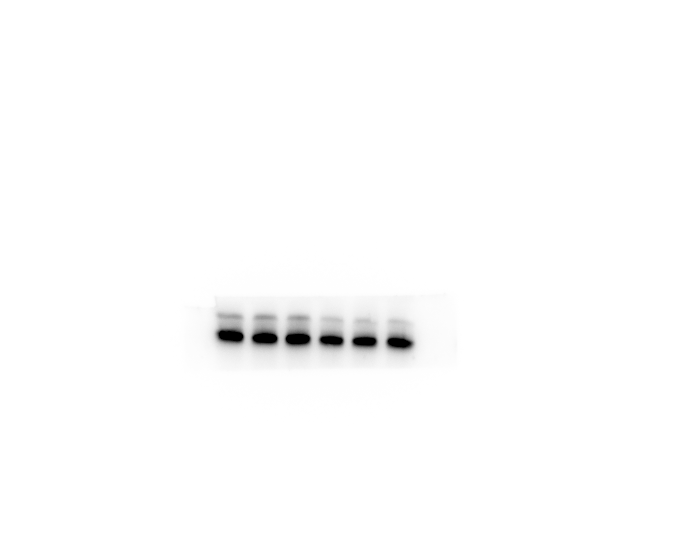

Supplement: Supplementary file 1 [file datasheet1.zip › The raw data of Western Blotting analysis/IκB/IκB-(3).tif]

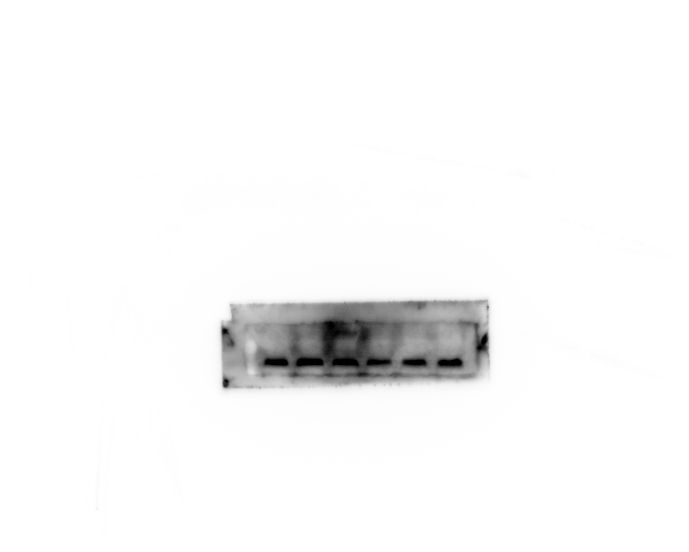

Supplement: Supplementary file 1 [file datasheet1.zip › The raw data of Western Blotting analysis/IκB/IκB-(4).tif]

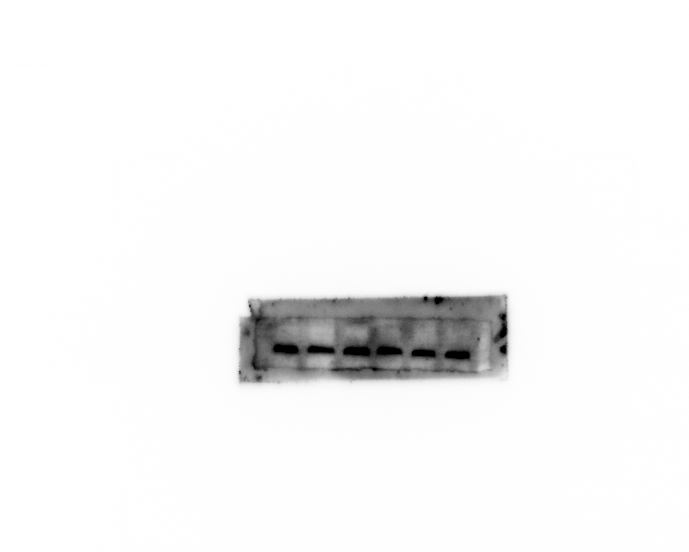

Supplement: Supplementary file 1 [file datasheet1.zip › The raw data of Western Blotting analysis/IκB/IκB-(5).tif]

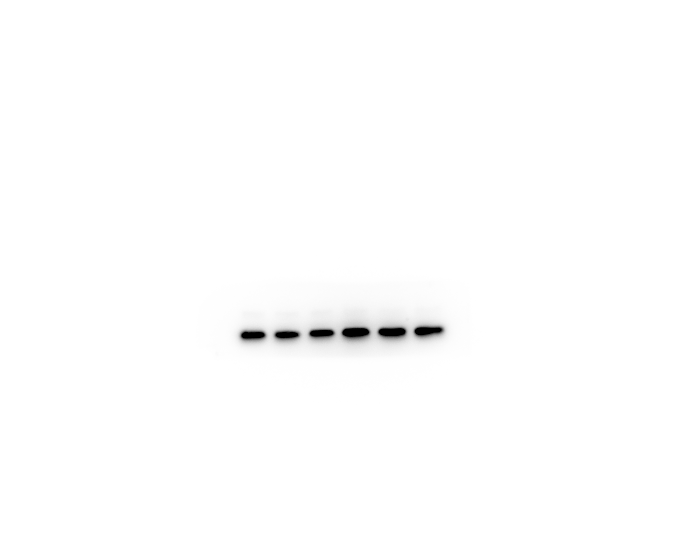

Supplement: Supplementary file 1 [file datasheet1.zip › The raw data of Western Blotting analysis/IκB/IκB-(6).tif]

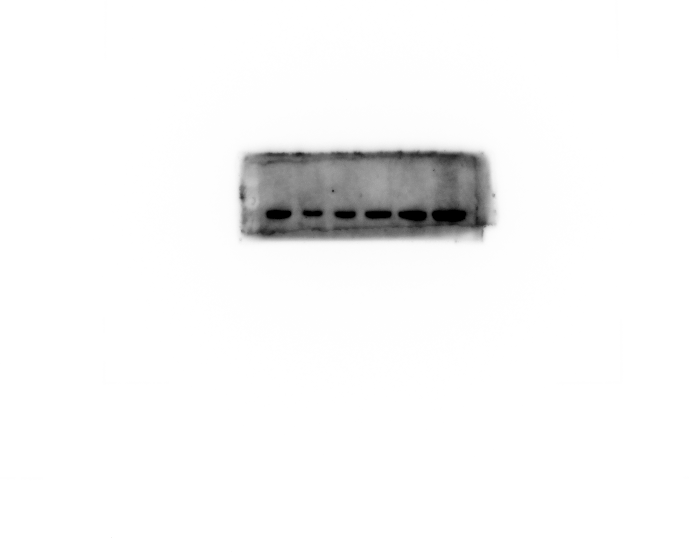

Supplement: Supplementary file 1 [file datasheet1.zip › The raw data of Western Blotting analysis/Nrf2/Nrf2(1).tif]

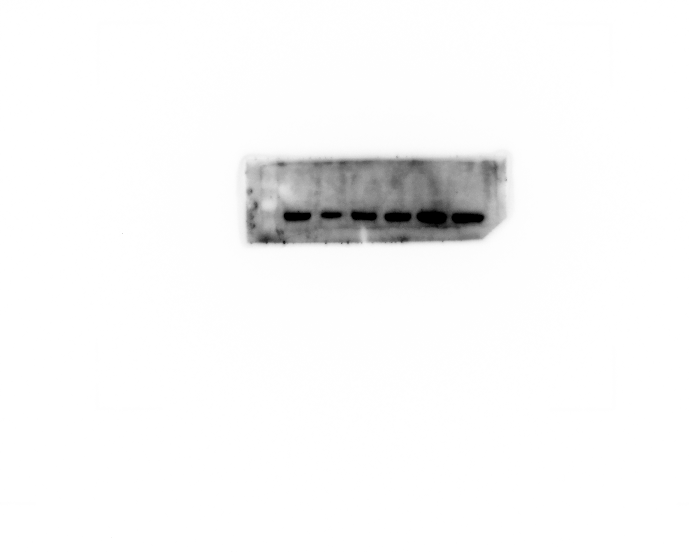

Supplement: Supplementary file 1 [file datasheet1.zip › The raw data of Western Blotting analysis/Nrf2/Nrf2(2).tif]

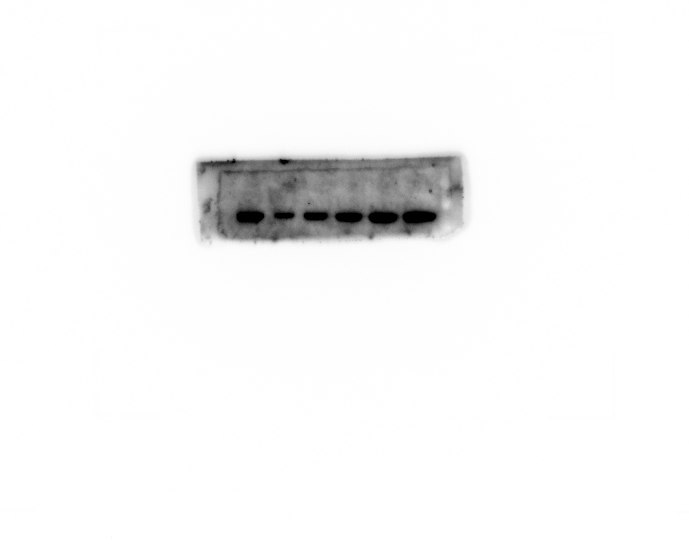

Supplement: Supplementary file 1 [file datasheet1.zip › The raw data of Western Blotting analysis/Nrf2/Nrf2(3).tif]

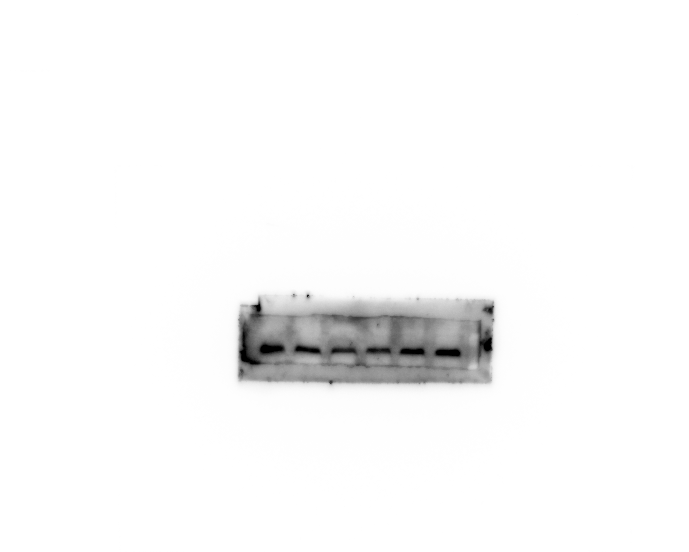

Supplement: Supplementary file 1 [file datasheet1.zip › The raw data of Western Blotting analysis/Nrf2/Nrf2(4).tif]

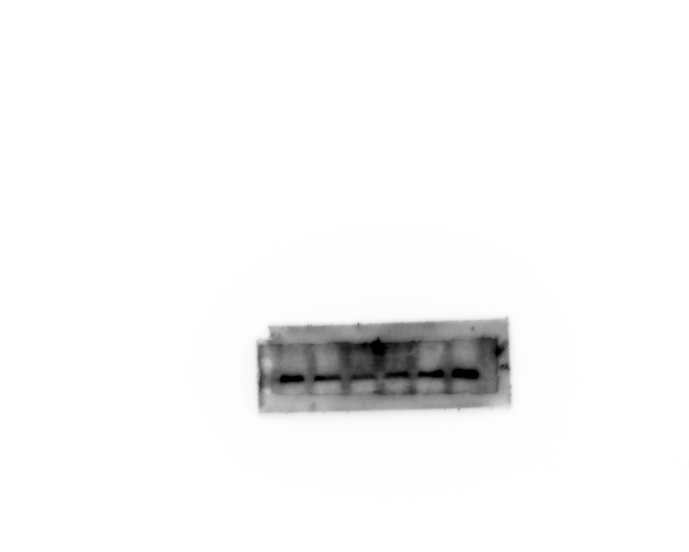

Supplement: Supplementary file 1 [file datasheet1.zip › The raw data of Western Blotting analysis/Nrf2/Nrf2(5).tif]

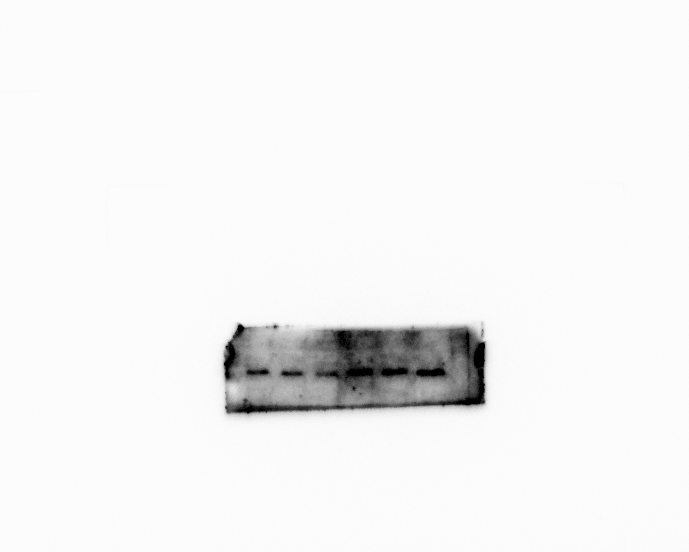

Supplement: Supplementary file 1 [file datasheet1.zip › The raw data of Western Blotting analysis/Nrf2/Nrf2(6).tif]

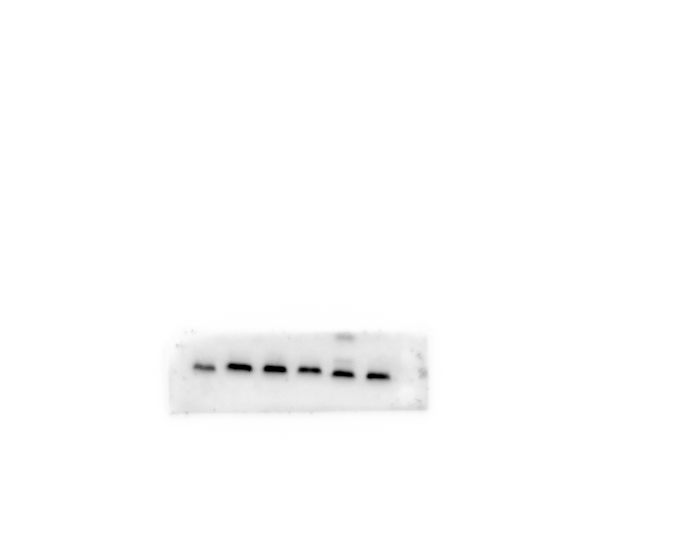

Supplement: Supplementary file 1 [file datasheet1.zip › The raw data of Western Blotting analysis/p-IκB/p-IκB(1).tif]

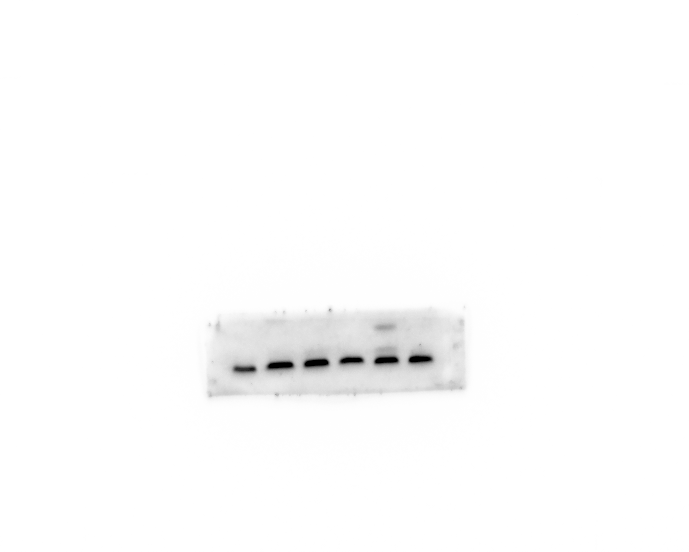

Supplement: Supplementary file 1 [file datasheet1.zip › The raw data of Western Blotting analysis/p-IκB/p-IκB(2).tif]

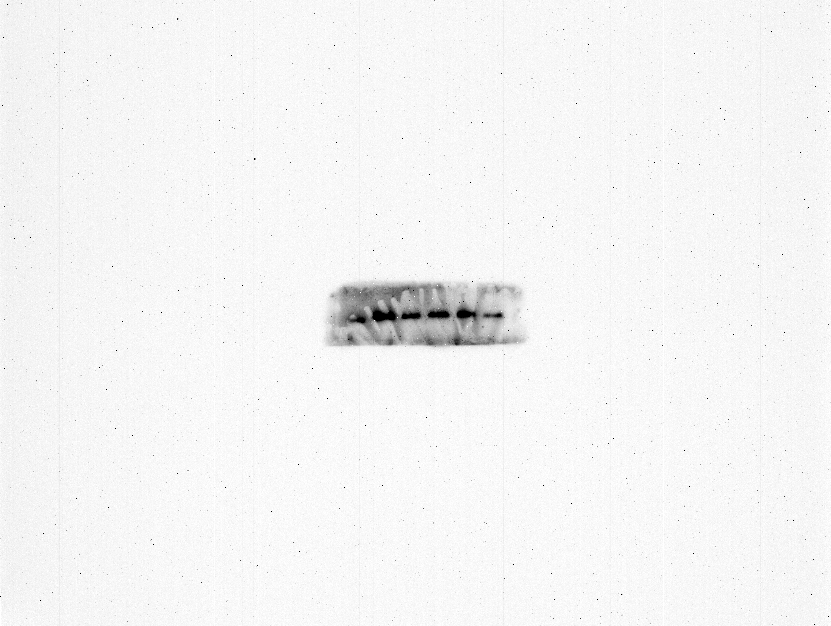

Supplement: Supplementary file 1 [file datasheet1.zip › The raw data of Western Blotting analysis/p-IκB/p-IκB(3).png]

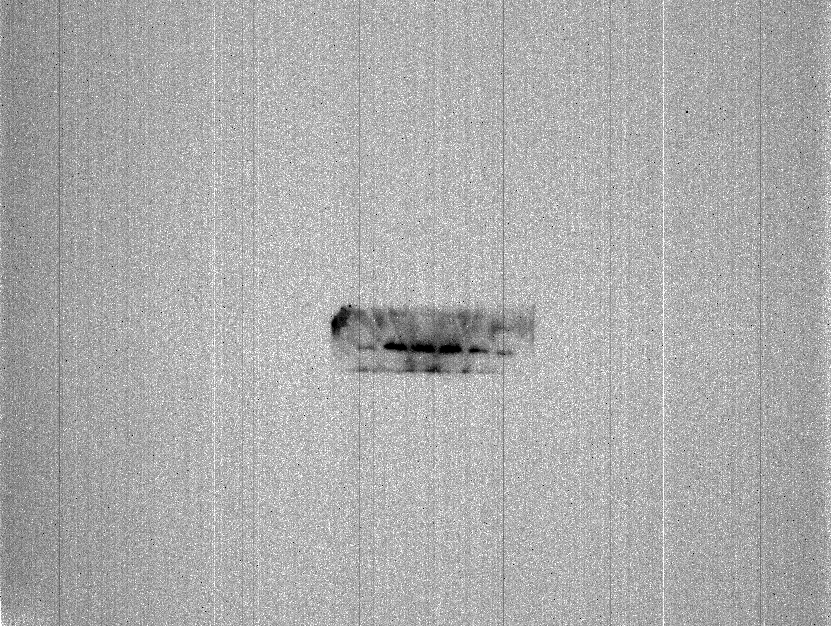

Supplement: Supplementary file 1 [file datasheet1.zip › The raw data of Western Blotting analysis/p-IκB/p-IκB(4).png]

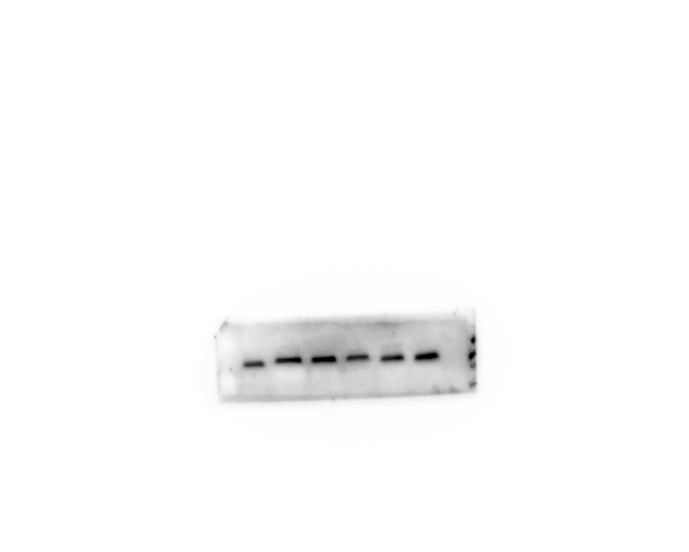

Supplement: Supplementary file 1 [file datasheet1.zip › The raw data of Western Blotting analysis/p-IκB/p-IκB(5).tif]

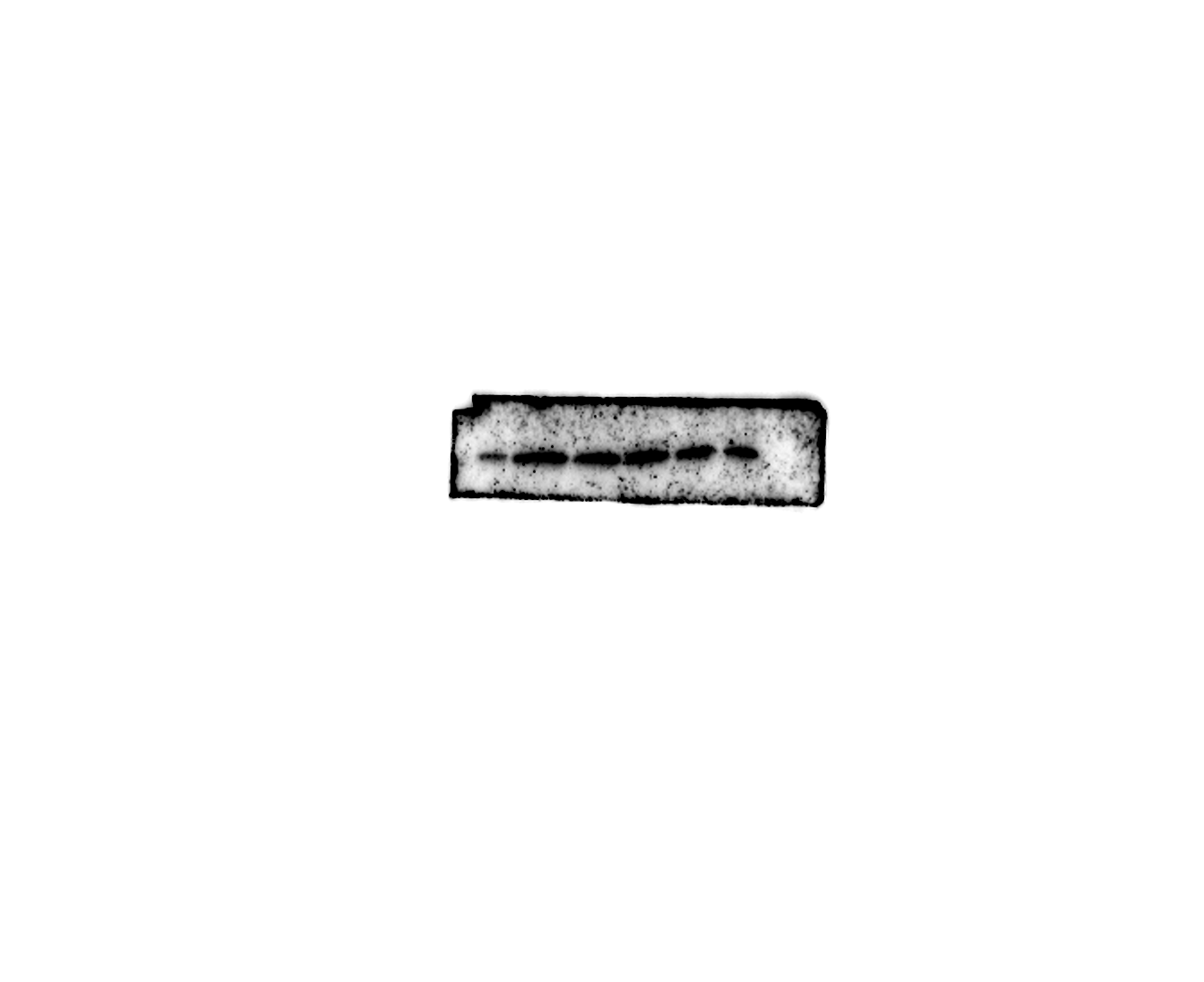

Supplement: Supplementary file 1 [file datasheet1.zip › The raw data of Western Blotting analysis/p-IκB/p-IκB(6).Tif]

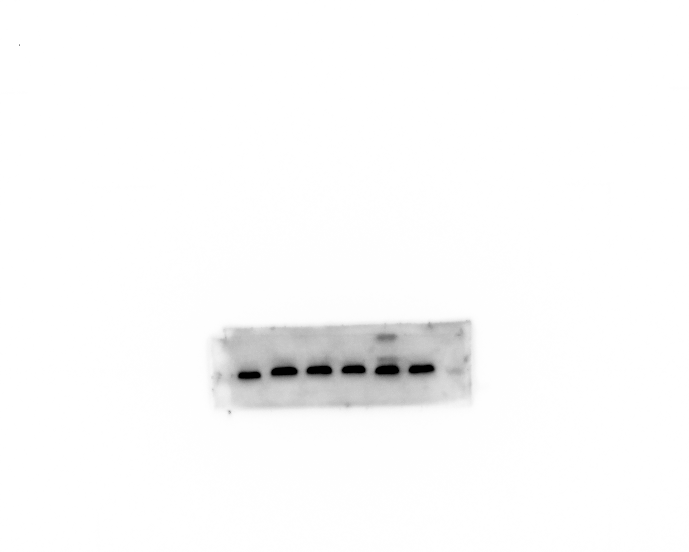

Supplement: Supplementary file 1 [file datasheet1.zip › The raw data of Western Blotting analysis/p-p65/p-p65(1).tif]

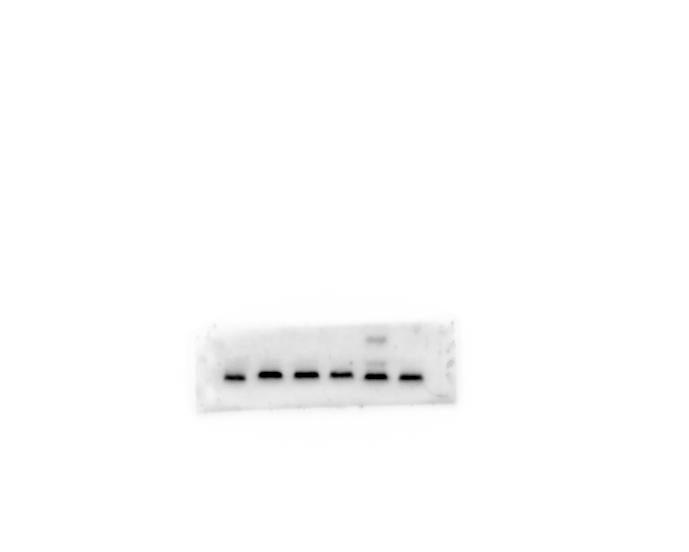

Supplement: Supplementary file 1 [file datasheet1.zip › The raw data of Western Blotting analysis/p-p65/p-p65(2).tif]

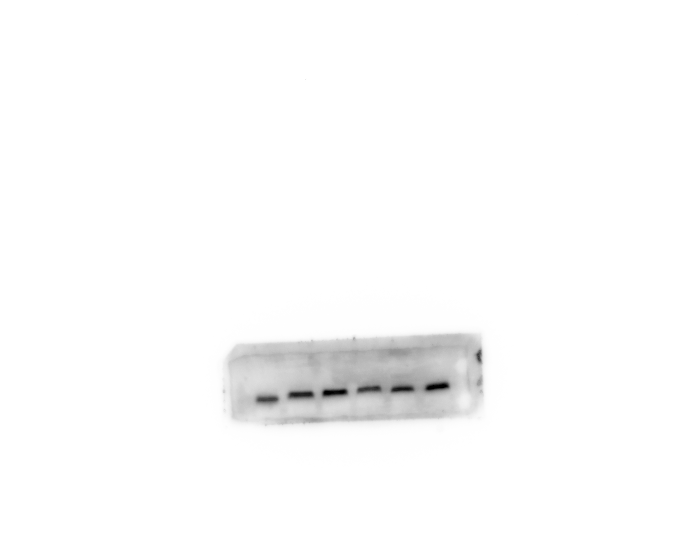

Supplement: Supplementary file 1 [file datasheet1.zip › The raw data of Western Blotting analysis/p-p65/p-p65(3).tif]

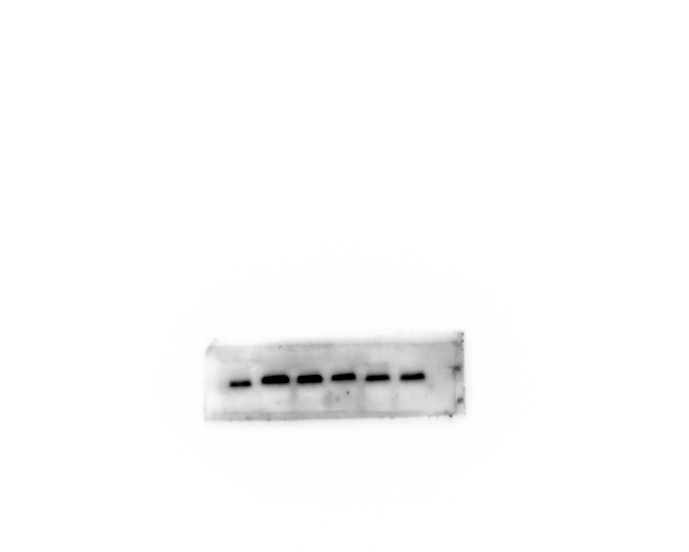

Supplement: Supplementary file 1 [file datasheet1.zip › The raw data of Western Blotting analysis/p-p65/p-p65(4).tif]

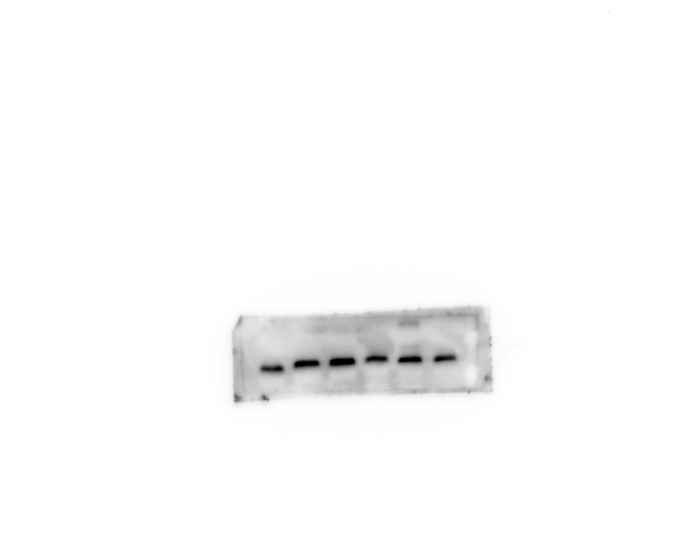

Supplement: Supplementary file 1 [file datasheet1.zip › The raw data of Western Blotting analysis/p-p65/p-p65(5).tif]

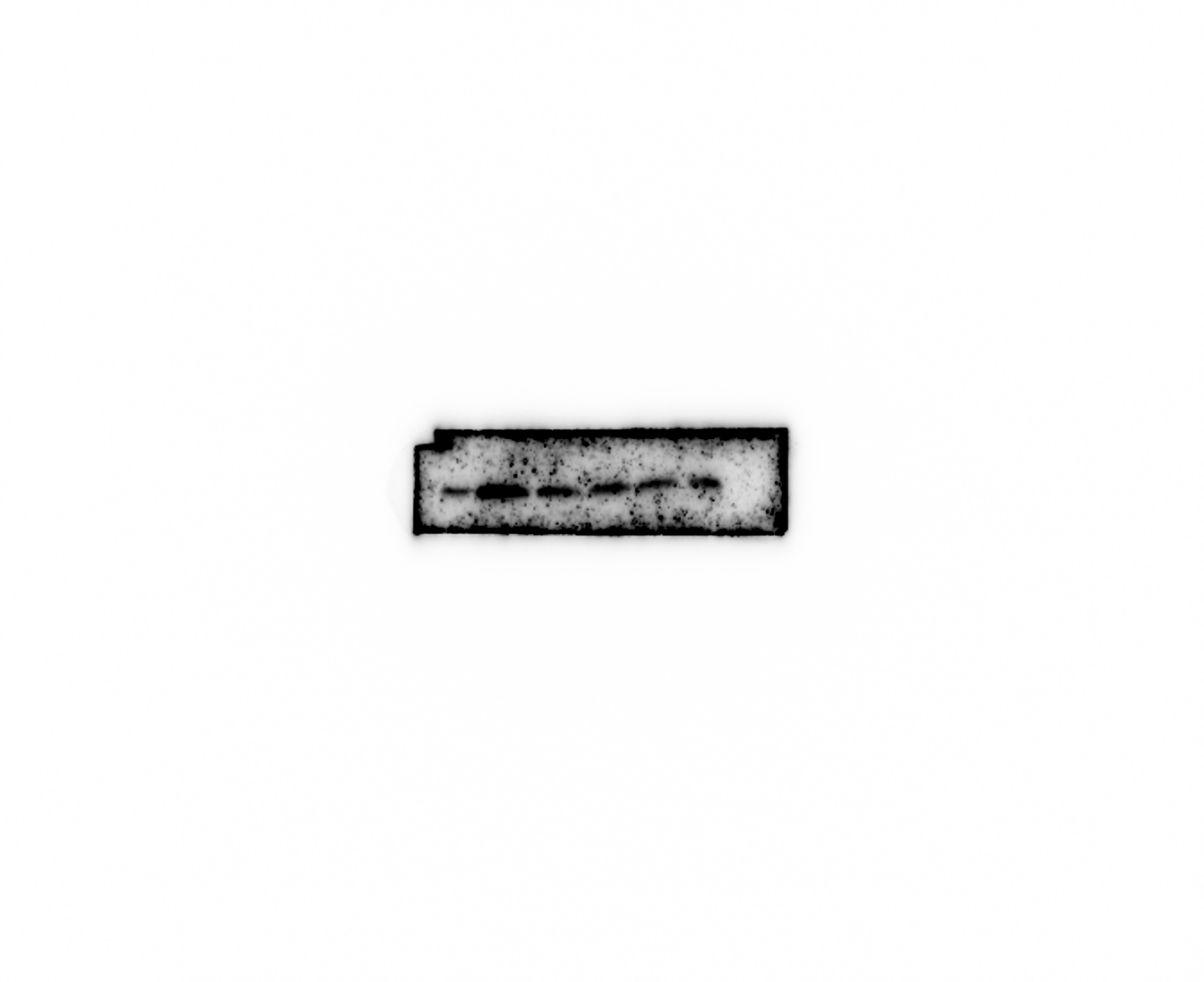

Supplement: Supplementary file 1 [file datasheet1.zip › The raw data of Western Blotting analysis/p-p65/p-p65(6).Tif]

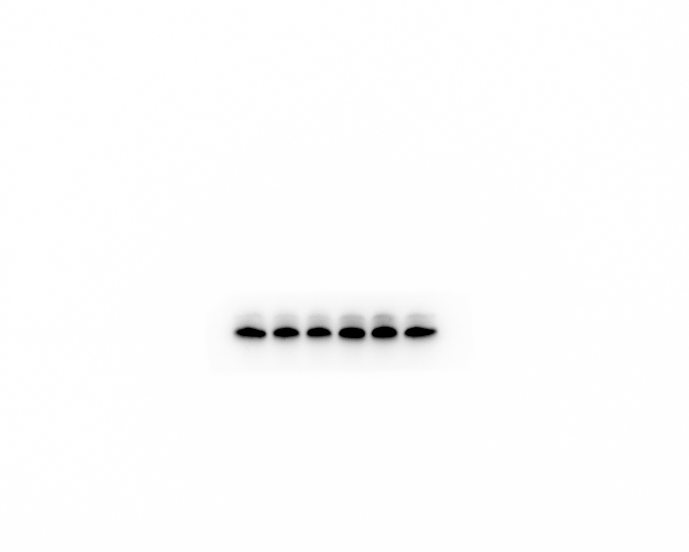

Supplement: Supplementary file 1 [file datasheet1.zip › The raw data of Western Blotting analysis/p65/p65(3).tif]

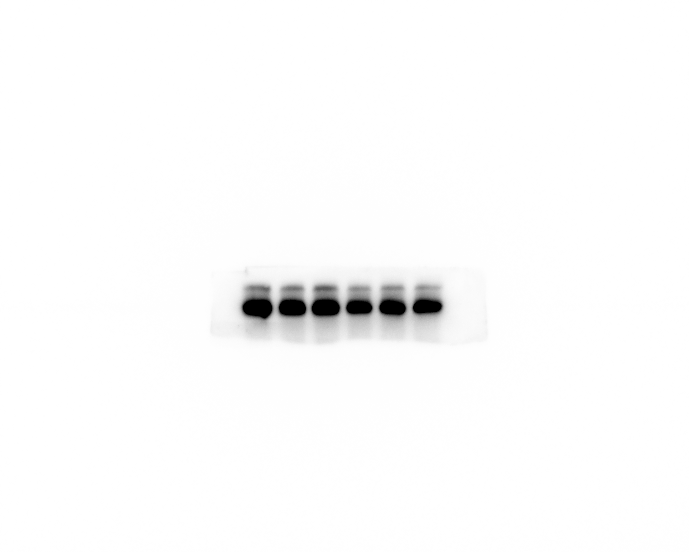

Supplement: Supplementary file 1 [file datasheet1.zip › The raw data of Western Blotting analysis/p65/p65(4).tif]

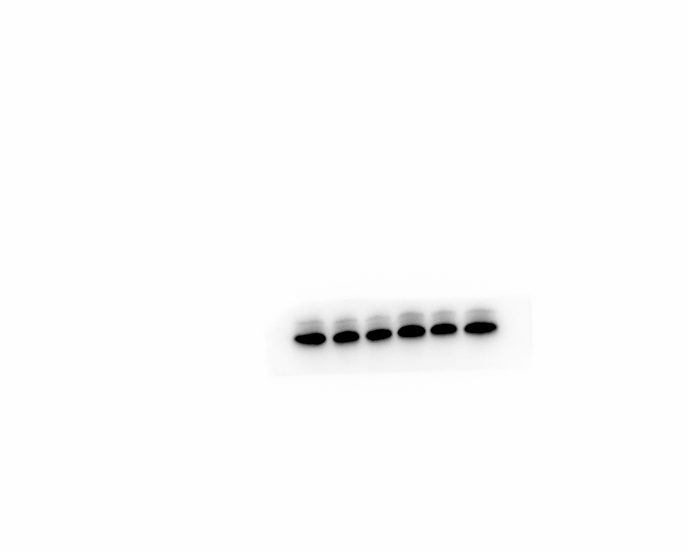

Supplement: Supplementary file 1 [file datasheet1.zip › The raw data of Western Blotting analysis/p65/p65(5).tif]

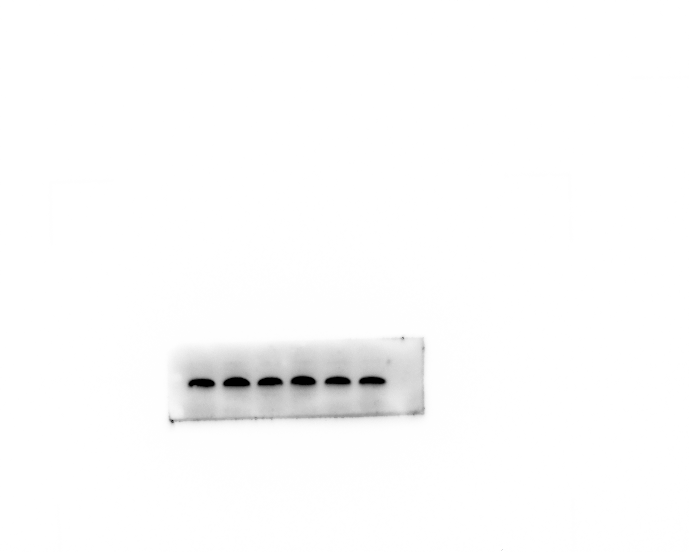

Supplement: Supplementary file 1 [file datasheet1.zip › The raw data of Western Blotting analysis/p65/p65(6).tif]

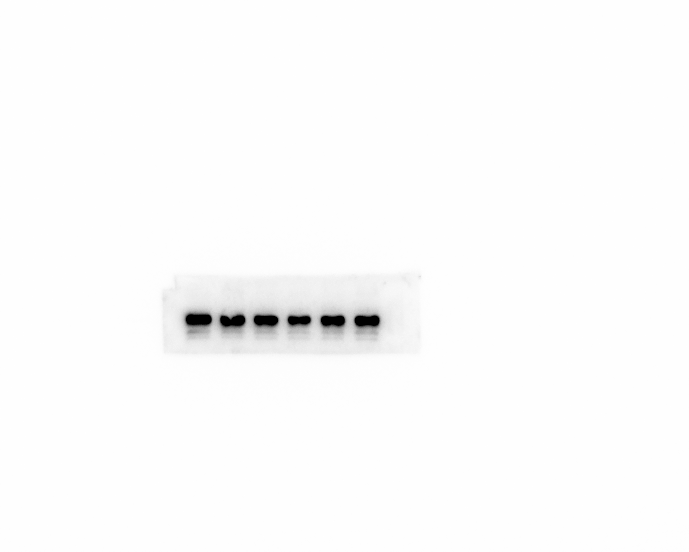

Supplement: Supplementary file 1 [file datasheet1.zip › The raw data of Western Blotting analysis/β-Actin/β-Actin(1).tif]

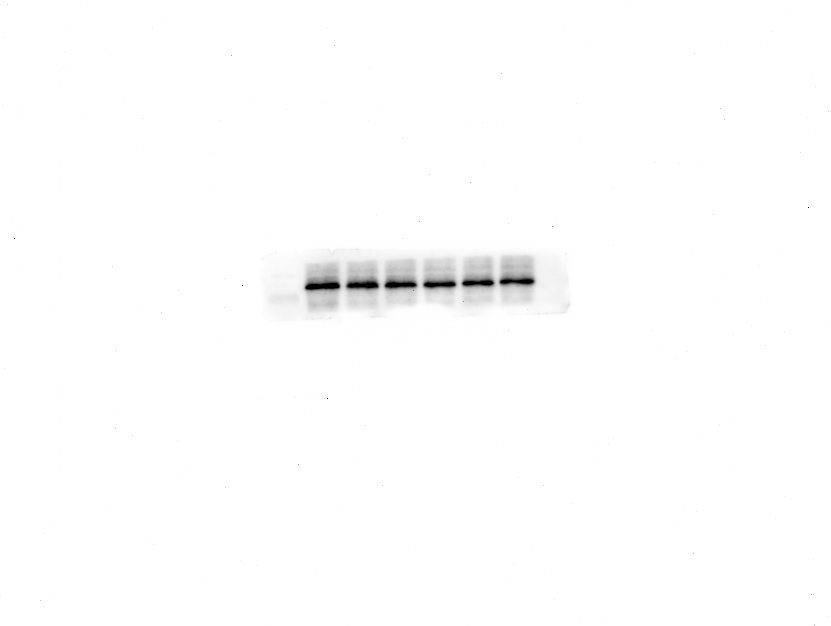

Supplement: Supplementary file 1 [file datasheet1.zip › The raw data of Western Blotting analysis/β-Actin/β-Actin(3).png]

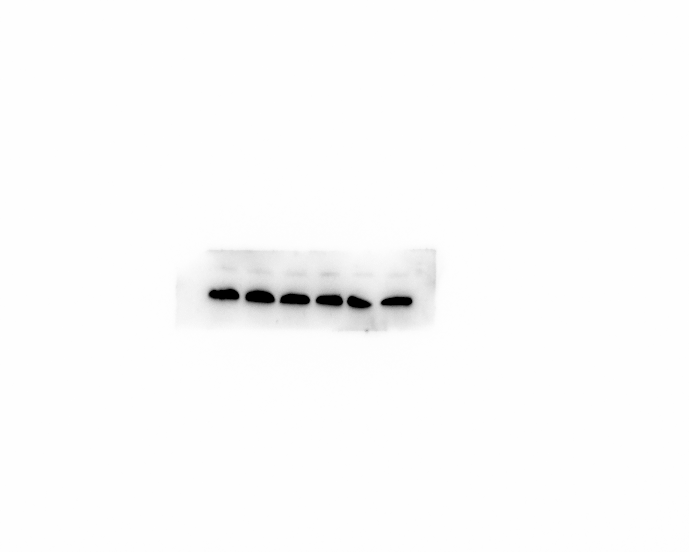

Supplement: Supplementary file 1 [file datasheet1.zip › The raw data of Western Blotting analysis/β-Actin/β-Actin(4).tif]

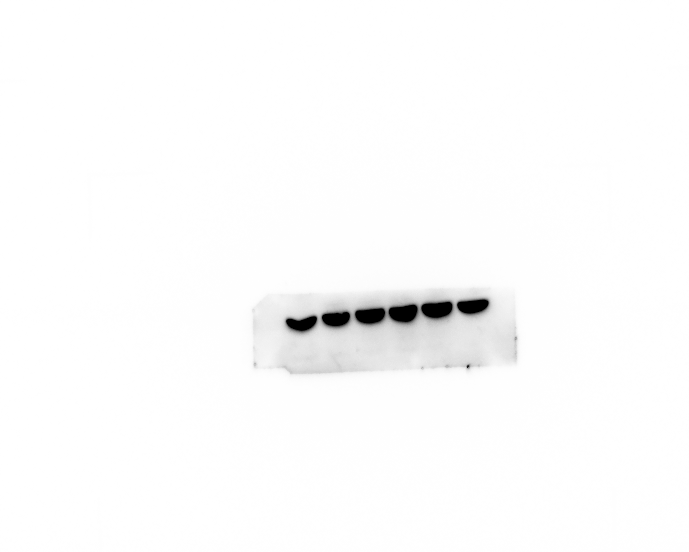

Supplement: Supplementary file 1 [file datasheet1.zip › The raw data of Western Blotting analysis/β-Actin/β-Actin(5).tif]

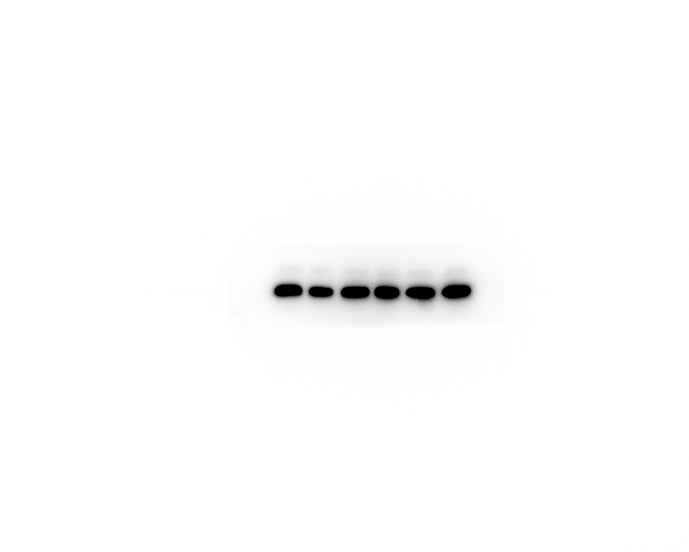

Supplement: Supplementary file 1 [file datasheet1.zip › The raw data of Western Blotting analysis/β-Actin/β-Actin(6).tif]
